# Extracellular Vesicles From *Xylella fastidiosa* Carry sRNAs and Genomic Islands, Suggesting Roles in Recipient Cells

**DOI:** 10.1002/jev2.70102

**Published:** 2025-06-25

**Authors:** Alessa Ruf, Patrick Blumenkamp, Christina Ludwig, Anne Lippegaus, Andreas Brachmann, Andreas Klingl, Alexander Goesmann, Karina Brinkrolf, Kai Papenfort, Silke Robatzek

**Affiliations:** ^1^ LMU Munich Biocenter Ludwig‐Maximilian‐University of Munich Martinsried Germany; ^2^ Bioinformatics and Systems Biology Justus Liebig University Giessen Giessen Germany; ^3^ Bavarian Center for Biomolecular Mass Spectrometry (BayBioMS) TUM School of Life Sciences Freising Germany; ^4^ Bioinstrumentezentrum Friedrich‐ Schiller University Institute of Microbiology Jena Germany; ^5^ Microverse Cluster Friedrich Schiller University Jena Germany

**Keywords:** EVs, genomic islands, Hfq, horizontal gene transfer, small noncoding RNAs

## Abstract

*Xylella fastidiosa* (*Xf*) is a Gram‐negative bacterial plant pathogen responsible for severe diseases in a variety of economically important crops. A critical aspect of its virulence is the production of extracellular vesicles (EVs). In this study, we discovered that DNA‐binding proteins and nonribosomal RNA‐binding proteins are abundant in the corona of *Xf*‐EVs. DNA‐seq revealed enrichment of three genomic islands (GIs) in EVs, which carry molecular signatures indicative of horizontal gene transfer (HGT). The most abundant GI encodes five homologous small RNAs designated *sXFs*. RNA sequencing revealed a distinct pattern of noncoding RNAs enriched in EVs, including four island‐encoded *sXFs*. One of the *sXF*’s stem‐loops contains motifs for binding the RNA chaperone Hfq, which is also abundant in EVs. Predicted target analysis suggests that *sXFs* play a role in regulation of natural competence in bacteria. Additionally, *sXF* plant target prediction identifies a coiled‐coil nucleotide‐binding domain leucine‐rich repeat receptor (*CNL*) immune gene that is downregulated following *Xf* infection and *Xf*‐EV treatment. We propose a model where *Xf* releases nucleic acid carrying EVs with two functions: one to deliver RNA‐related cargo that regulates gene expression in both bacterial and plant cells, and another to deliver DNA‐related cargo for the genetic transfer of genomic islands. We highlight island‐encoded *sXFs* as potential virulence factors and vesiduction as a mechanism of HGT of *sXFs* in *Xf*. Taken together, our data on *Xf*‐EV cargoes provide a molecular framework for understanding the virulence of *Xf*.

## 1 Introduction

Pathogenic bacteria must adapt to and modulate their host's environment to ensure their survival and the establishment of infection within the host. Gram‐negative bacteria release extracellular vesicles (EVs) to facilitate their proliferation, for example, by detoxifying host defences, immune suppression and nutrient acquisition as well as competing and cooperating with strains of host‐associated bacterial communities (Rybak and Robatzek 2019; McMillan and Kuehn 2021; Toyofuku et al. 2023). EVs are mobile cytosol‐derived cargo containing nanosized membrane spheres that are produced by bacteria in the form of outer membrane vesicles (OMVs) and outer inner membrane vesicles (OIMVs) (Toyofuku et al. 2023). Different biogenesis routes of EVs indicate the release of heterogenic subpopulations of EVs with different types of cargo (Toyofuku et al. 2023). As long as biomarkers are not available to convincingly probe their origin, we will collectively refer to these vesicles as EVs.

EVs are classified as a bacterial Type‐0 secretion system, selectively delivering bacterial cargoes into the environment. The cargoes include proteins, and nucleic acids present at the vesicle corona, a layer of biomolecules adsorbed onto the vesicle surface—as well as the membrane or the lumen, which provides protection against degradation. Since the EV cargo composition responds to developmental and environmental conditions, vesiculation enhances bacterial adaptability (Xiu et al. 2024). For example, EVs can carry toxins and enzymes that attack and degrade the cell walls of competitor strains or hosts, thus providing an advantage to the vesiculating bacteria (Toyofuku et al. 2023; Macion et al. 2021; De La Fuente et al. 2022). Moreover, EVs interact with recipient cells by delivering cargo to surface receptors or into the cytoplasm, leading to altered cellular processes in the recipient cells. This includes the modulation of host immune signalling by EV‐packaged small (s)RNAs, which aids the bacterial infection process (Koeppen et al. 2016; Wu et al. 2024; Xie et al. 2024).

EVs are relevant for interbacterial competition and cooperation and facilitate the transfer of genetic material, including plasmids and antibiotic resistance genes, thereby enhancing genetic diversity and adaptability within microbial communities. EVs also enable horizontal gene transfer (HGT) among bacteria through a process known as vesiduction (Marinacci et al. 2023; Soler and Forterre 2020; Dell'Annunziata et al. 2021). In this process, genetic material is encapsulated within EVs, and upon delivery to recipient cells, it can integrate into the recipient's genome, leading to genetic transformation. Vesiduction is a crucial mechanism for the spread of antibiotic resistance and virulence traits, as it promotes the rapid dissemination of genetic information across bacterial populations and even across species barriers (Marinacci et al. 2023; Soler and Forterre 2020; Li et al. 2022; Johnston et al. 2023; Wang et al. 2022; Qiao et al. 2021). Since EVs protect DNA from degradation during transfer, vesiduction increases the efficiency of HGT, aiding bacterial evolution and adaptation (Marinacci et al. 2023). Canonical secretion systems, such as the Type‐VI secretion system in *Cupriavidus necator*, function together with EVs in HGT by secreting molecules, which bind to lipopolysaccharides (LPSs) on EVs and tethers them to recipient cells (Li et al. 2022).

*Xylella fastidiosa* (*Xf*), a Gram‐negative bacterium in the Xanthomonadaceae family, is a plant pathogen listed as a ‘priority pest’ in Europe. *Xf* exclusively colonizes two habitats: the plant xylem vessels and the foregut of insect vectors, such as sharpshooter leafhoppers and spittlebugs (Castro et al. 2021). Transmitted by these xylem sap‐feeding insects, the bacterium infects around 700 plant species (EFSA 2023), causing severe agricultural diseases. These include Pierce's disease (PD) in grapevines caused by *X. fastidiosa* subsp*. fastidiosa* (*Xff*), as well as Citrus Variegation Chlorosis (CVC) in citrus trees and the Olive Quick Decline Syndrome (OQDS) in olives triggered by *X. fastidiosa* subsp*. pauca* (*Xfp*) (Landa et al. 2022). With its genome sequence serving as a reference and its genetic tractability, *Xff* strain Temecula1 (*Xff* Tem1) has provided most molecular mechanistic insights into *Xf* (Landa et al. 2022). The *Xfp* strain De Donno (*Xfp* DD) has a significant agricultural impact and is responsible for the recent outbreak of OQDS in Southern Europe (Landa et al. 2022). Because these two strains are adapted to different hosts, they offer a valuable opportunity to address the immune evasion modes of *Xf*.

The nonflagellated *Xf* bacteria spread through the xylem via both passive flow and twitching motility (Rapicavoli et al. 2018). As bacterial density increases, biofilm formation is initiated. The shift from planktonic to sessile states is regulated by quorum sensing (QS) and is driven by EVs, which promote bacterial detachment from surfaces (Ionescu et al. 2014). The link between vesiculation and surface detachment is evident from the genetic deletion of a diffusible signal factor (DSF) synthase, *rpfF*, in *Xff* Tem1, which led to hypervesiculation. This increased vesicle production reduced the bacterium's ability to attach to surfaces, thereby enhancing its ability to spread systemically (Ionescu et al. 2014). Previous proteomic analyses of EV‐enriched fractions from cultured *Xf* revealed adhesins as cargoes, further supporting this phenotypic observation (Feitosa‐Junior et al. 2019). Comparative analysis of EVs from *Xff* Tem1 and *Xff* DD provides insights into shared cargo that could influence their ability to colonize hosts.

The presence of cell wall‐degrading enzymes suggests that *Xf* EVs contribute to the bacterium's virulence by promoting its systemic colonization, likely access to the living cells within the xylem tissue (Nascimento et al. 2016; Roper et al. 2007). Since cell wall degradation can release damage‐associated molecular patterns (DAMPs), it is likely that this process triggers immune signalling (Lorrai and Ferrari 2021). Moreover, *Xf*‐EVs carry the translation elongation factor EF‐Tu, which induces plant immunity (Feitosa‐Junior et al. 2019; Mitre et al. 2021; Nascimento et al. 2016). To evade host immunity, *Xff* Tem1 has been shown to shield its LPS (Rapicavoli et al. 2018). However, *Xf* lacks a Type‐III secretion system, by which Gram‐negative bacteria typically deliver Type‐III‐secreted effectors to suppress host defences and reprogram plant physiology (Macho and Zipfel 2015). This raises the question of how *Xf* overcomes plant immune responses induced by EF‐Tu, the cold shock protein peptide csp22 and cell wall damage, for example, vessel lignification (Sabella et al. 2018; Burbank and Ochoa 2022).

Given the role of EVs in bacterial pathogenesis and as a Type‐0 secretion system (Guerrero‐Mandujano et al. 2017), we speculate that *Xf* utilizes EVs as a mechanism for delivering effectors in a contact‐independent manner. To address this hypothesis, we profiled size‐exclusion chromatography (SEC)‐purified *Xf*‐EV cargoes by proteomics, DNA sequencing (DNA‐seq) and RNA sequencing (RNA‐seq). Our analysis revealed that EVs have distinct protein, DNA and RNA profiles compared to whole‐cell lysates (WCLs). EVs were enriched with noncoding (nc)RNAs, including four homologous small (s)RNAs, designated *sXFs*, which are encoded on a genomic island (GI) that is also enriched in EVs as DNA. Notably, a *sXF* predicted plant target is downregulated in *Xff*‐infected and *Xff*‐EV‐treated plants.

## 2 Material and Methods

### 2.1 Culturing of *Xylella fastidiosa*

*Xff* Tem1 and *Xfp* DD were routinely grown for 5–10 days on Pierce's disease 3 (PD3) plates (Davis 1980). For liquid cultures, 200 mL PD2 (Davis 1980) was inoculated with resuspended *Xf* inoculum in phosphate buffered saline (PBS), and cultures were grown for approx. 4–7 days in PD2 (+50 µg/mL for Temecula1‐GFP) at 28°C and 140 revolutions per minute (rpm), reaching an OD_600_> 0.2.

### 2.2 EV Isolation and Purification

Before harvesting cells, cultures were shaken to collect all cells from the cultures, including biofilm and planktonic cells in suspension. For WCL samples, 2 mL cultures were centrifuged for 15 min, 4000 × *g*. Supernatants were removed, and pellets were flash‐frozen and kept at −80°C until further use. For *Xf*‐EV isolation, 200 mL cultures were centrifuged at 4000 × *g* for 15 min, supernatants were filtered through 0.22 µm filters (Milipore, SEGTPT0045). Filtered supernatants were centrifuged at 38,000 × *g* for 1 h to remove cellular debris and then further centrifuged at 150,000 × *g* for 4 h. Then, pellets were resuspended in 2 mL filtered 1× PBS (pH 7.4) and further purified using qEV2 iZON SEC‐columns (iZon qEV2 columns, 70 nm series, IC2‐70, France). Fractions containing *Xf*‐EVs were collected and either enriched using the qEV Concentration Kit (iZon; RCT02, France), qEV Magnetic Concentration Kit (iZon; RCT03, France) or Amicon centrifugal columns 30 kDa (Milipore, UFC9030), depending on further usage. An overview of the *Xf*‐EV isolation workflow can be found in Figure S1A. For nucleic acid analysis, *Xff* Tem1‐EVs were isolated from 1× 200 mL (DNA) or 2× 200 mL (RNA) cultures and concentrated using iZon concentrator beads (for DNA) or iZon magnetic concentrator beads (for RNA).

### 2.3 EV Enzyme Treatments and Disruption

For enzyme treatments, SEC‐purified *Xff* Tem1‐EV samples (8 mL) were split into five fractions. Fraction 1 (untreated) was kept at 4°C while Fractions 2 and 3 were treated with 100 µg/mL Proteinase K (ProtK, 800U, NEB, P81075) for 30 min at 37°C. The reaction was stopped by addition of 6.6 µL of 100 mM PMSF and incubation at RT for 5 min. Then, Fraction 3 was treated with 10,000 U Micrococcal Nuclease (MNase, NEB M0247S) for 30 min at 37°C.

Fraction 4 was used for disruption assays of EVs with 1% Triton were performed according to (Huang et al. 2021). Fraction 5 was used for EV‐burst assays SEC‐purified EVs were washed twice with hypotonic buffer (2 mM Tris‐HCl, 1 mM MgCl_2_, 1 mM KCl) (Cheng et al. 2018) in Amicon centrifugal columns 30 kDa (Milipore, UFC5003), followed by ProtK and MNase treatments as described in 2.3. All fractions were incubated with 100 µL of qEV Magnetic concentrator beads (iZon; RCT03, France) at RT for 10 min and put on a magnetic stand to remove supernatant, following manufacture's description. Next, concentrated *Xff* Tem1‐EV pellets were used for DNA or RNA extraction as described in 2.11 and 2.13.

### 2.4 SEM and TEM Analysis

For scanning electron microscopy (SEM), samples were prepared as described previously (Janda et al. 2023). Microscopy was carried out on a Zeiss Auriga Crossbeam workstation (Zeiss, Oberkochen, Germany) at an acceleration voltage of 1.5 kV and a working distance of approximately 5 mm and by using the secondary electron (SE) detector. For transmission electron microscopy (TEM), isolated EVs were negatively stained with 1% uranyl acetate. Microscopy was carried out at 200 kV using a JEOL F200 (JEOL, Japan), equipped with a 20 mega pixel CMOS camera.

### 2.5 Nanoparticle Tracking Analysis (NTA)

For EV characterization using a nanoparticle tracking analyser (ZetaView, Particle Metrix, Germany), appr. 10 µL of SEC‐purified *Xf*‐EVs were kept before further concentration with iZon concentrator beads. *Xf*‐EVs were diluted to a concentration which resulted in approx. 200–300 particles per window; vesicle size and charge (zeta [*ζ*] potential) were determined with three measurements per sample.

### 2.6 Labelling of *Xf*‐EVs

For labelling of *Xf*‐EVs, 100 µL of isolated vesicles were incubated with 1 mM FM4‐64 (Invitrogen, T13320) and 1 mM SYTO RNAselect (Invitrogen, S32703) at room temperature (RT) for approx. 20 min. Then, any unincorporated dye was removed using Amicon centrifugal columns 30 kDa (Milipore, UFC5003), and columns were washed with 3× 100 µL 1× PBS. Signals were imaged using Leica Thunder imager with 100×/1.44 OIL UV objective. FM4‐64 was excited at 390 nm and signal was collected over the whole spectrum. RNAselect was excited at 510 nm and the signal was collected at an emission wavelength 535 nm.

### 2.7 Coomassie Staining

To estimate protein concentrations, Bradford assays were performed following the microassays (Sigma–Aldrich, B6916) according to the manufacturer's guidelines (Figure S2a, b). 30 µL of prepared samples were loaded onto 12% sodium dodecyl sulphate (SDS)‐polyacrylamide gel electrophoresis and protein profiles were checked by colloidal Coomassie staining overnight.

### 2.8 Sample Preparation for Proteomics

SEC‐purified *Xf*‐EV samples (8 mL) were split into two fractions. Fraction 1 (untreated) was kept at 4°C while Fraction 2 (ProtK treated) was treated with 100 µg/mL ProtK (800U, NEB, P81075) for 30 min at 37°C. The reaction was stopped by the addition of 6.6 µL of 100 mM PMSF and incubation at RT for 5 min. Then, both samples were incubated with 100 µL of iZon concentrator beads at RT for 1 h and pelleted at 16,800 × *g* for 10 min. Pellets were resuspended in 100 µL 1× PBS (pH 7.4). After the addition of 20 µL 6× Lämmli buffer 9.5 µL 50 mM dithiothreitol (DTT), samples were boiled for 10 min at 98°C and then stored at −80°C until further use.

Four biological replicates of WCL, *Xf*‐EV and *Xf*‐EV + ProtK samples from *Xff* Tem1 as well as *Xfp* DD were denatured by the addition of 1× SDS loading buffer and heated for 10 min at 70°C. In‐gel trypsin digestion was performed according to standard procedures (Shevchenko et al. 2006). In order to ensure equal protein concentrations across all analysed samples, a consistent protein amount of 10 µg per sample was run on a Nu‐PAGE 4%–12% Bis‐Tris protein gel (ThermoFisher Scientific) for about 1 cm. Subsequently, the still not size‐separated single protein band per sample was cut, reduced (50 mM DTT), alkylated (55 mM chloroacetamide) and digested overnight with trypsin (Trypsin Gold, mass spectrometry grade, Promega). The obtained peptides were dried to completeness and resuspended in 24 µL (WCL) or 12 µL (*Xf*‐EV and *Xf*‐EV + ProtK) of 2% acetonitrile, 0.1% formic acid (FA) in HPLC grade water. Finally, 2 µL (WCL) or 5 µL (*Xf*‐EV and *Xf*‐EV + ProtK) of the sample was injected per mass spectrometric (MS) measurement.

### 2.9 Liquid Chromatography‐Mass Spectrometry (LC‐MS/MS) Data Acquisition

LC‐MS/MS data acquisition was carried out on a Dionex Ultimate 3000 RSLCnano system coupled to a Q‐Exactive HF‐X mass spectrometer (ThermoFisher Scientific, Bremen). Injected peptides were delivered to a trap column (ReproSil‐pur C18‐AQ, 5 µm, Dr. Maisch, 20 mm × 75 µm, self‐packed) at a flow rate of 5 µL/min in 0.1% FA in HPLC grade water. After 10 min of loading, peptides were transferred to an analytical column (ReproSil Gold C18‐AQ, 3 µm, Dr. Maisch, 450 mm × 75 µm, self‐packed) and separated using a 50 min gradient from 4% to 32% of Solvent B (0.1% FA, 5% DMSO in acetonitrile) in Solvent A (0.1% FA, 5% dimethyl sulphoxide [DMSO] in HPLC grade water) at 300 nL/min flow rate. The Q‐Exactive HF‐X mass spectrometer was operated in data‐dependent acquisition (DDA) and positive ionization mode. MS1 spectra (360–1300 *m*/*z*) were recorded at a resolution of 60,000 using an automatic gain control (AGC) target value of 3e6 and a maximum injection time (maxIT) of 45 ms. Up to 18 peptide precursors were selected for fragmentation. Only precursors with a charge state of 2–6 were selected, and dynamic exclusion of 25 s was enabled. Peptide fragmentation was performed using higher energy collision‐induced dissociation (HCD) and a normalized collision energy (NCE) of 26%. The precursor isolation window width was set to 1.3 *m*/*z*. MS2 Resolution was 15,000 with AGC target value of 1e5 and maxIT of 25 ms.

### 2.10 LC‐MS/MS Data Analysis

Peptide identification and quantification was performed using the software MaxQuant (version 1.6.3.4) (Tyanova et al. 2016) with its built‐in search engine Andromeda (Cox et al. 2011). MS2 spectra were searched against either the Uniprot protein database of *Xf* strain Temecula1 (ATCC 700964, UP000002516, 2007 protein entries, downloaded August 2023) or the Uniprot protein database of *Xfp* (UP000194146, 2059 protein entries, downloaded August 2023), respectively. The databases were further supplemented with the amino acid sequence of ProtK from *Parengyodontium album* (P06873) as well as common contaminants (built‐in option in MaxQuant). Trypsin/P was specified as proteolytic enzyme. Carbamidomethylated cysteine was set as a fixed modification. Oxidation of methionine and acetylation at the protein N‐terminus was specified as variable modifications. Results were adjusted to 1% false discovery rate (FDR) on the peptide spectrum match (PSM) level and protein level employing a target‐decoy approach using reversed protein sequences. Label‐free quantification (LFQ) (Cox et al. 2014) intensities were used for protein quantification with at least two peptides per protein identified. The minimal peptide length was defined as seven amino acids, and the ‘match‐between‐runs’ functionality was disabled. Only proteins for which LFQ values in at least three out of four replicates in at least one of the sample types were considered for further analysis. Missing values were imputed by a protein‐specific constant value, which was defined as the lowest detected protein‐specific LFQ‐value over all samples divided by two. Additionally, a maximal imputed LFQ value was defined as the 15% quantile of the protein distribution from the complete dataset.

To identify proteins with a significantly higher abundance in EV samples compared to WCL samples, a Welch's *t* test was conducted. The resulting *p* values were corrected using the Benjamini–Hochberg method to control the FDR. Proteins with FDR < 0.05 and a fold‐change > 1 in *Xf*‐EVs compared to WCL were categorized as ‘EV‐enriched proteins’. All other proteins detected in the *Xf*‐EV (or *Xf*‐EV + ProtK) samples in at least three out of four replicates were categorized as ‘present in EVs’. For functional categorization, identified EV proteins were manually sorted. Gene ontology (GO)‐term enrichment was performed on all identified EV proteins, using an adjusted *p* value cutoff < 0.05. For rank analysis, (imputed) expression values for all EV proteins were averaged over the four replicates and ranked from highest to lowest. To compare the proteomes of both *Xf* subspecies, we reanalysed the *Xfp* DD data files against the *Xff* Tem1 reference database (*Xf strain* Temecula1, ATCC 700964, UP000002516). Proteins identified in both subspecies in at least three out of four replicates were defined as ‘core EV cargo’. Functional categorization was also achieved through manual sorting. For comparison of identified EV proteins with previous publications (Feitosa‐Junior et al. 2019; Nascimento et al. 2016), the presence of EV‐protein content was compared using GeneID or ProteinIDs, respectively.

### 2.11 DNA Isolation and DNA Sequencing

DNA isolation was performed using phenol/chloroform extraction as published in Spada et al. (2020). EV pellets were resuspended in DNA extraction buffer (SDS 0.5%, Tris‐HCL 50 mM pH 8, EDTA 0.1 M) and 0.1 mg/mL of ProtK and incubated at 56°C overnight. Then, exosomal DNA was isolated using phenol/chloroform. DNA was pelleted for 2 h at −80°C and precipitation was facilitated by the addition of 1 µL glycogen (Thermo Scientific, FERR0051). DNA of untreated *Xff* Tem1‐EVs was then used to perform DNA sequencing. The size and concentration of DNA were analysed using DNA high‐sensitivity bioanalyzer chips. Sequencing libraries were constructed from 1 ng of DNA with the Nextera XT DNA Sample Preparation Kit (Illumina, Germany) according to the manufacturer's protocol. The library was quality controlled by analysis on an Agilent 2000 Bioanalyzer with the Agilent High Sensitivity DNA Kit (Agilent Technologies, Germany) for fragment sizes of ca. 500–800 bp. Sequencing on a MiSeq sequencer (Illumina; 2× 300 bp paired‐end sequencing, v3 chemistry) was performed in the Genomics Service Unit (LMU Biocenter, Martinsried, Germany). For PCR amplification of GI genes, EV pellets from Fractions 1–5 were used for DNA extraction, followed by regular PCR amplification using gene‐specific primers and GoTaq (Promega, M7122) with 45× amplification cycles.

### 2.12 DNA Analysis

The DNA sequences were adapter and quality trimmed with fastp v.0.23.4 (Chen et al. 2018) using default settings without length filtering (‘–disable_length_filtering’). Due to a larger region of badly aligned reads in rRNA regions, DNA reads of rRNAs were filtered out with SortMeRNA v.4.3.6 (Kopylova et al. 2012) using ‘smr_v4.3_default_db.fasta’ from https://github.com/biocore/sortmerna/releases/download/v4.3.4/database.tar.gz. Afterwards, the reads were aligned to NCBI accession GCF_000007245.1 with Bowtie 2 v.2.5.3 (Langmead and Salzberg 2012) in ‘–very‐sensitive’ mode and a maximal insert size of 1000 bp (−X 1000). Conversion, sorting and filtering (unmapped reads) of SAM/BAM files were done with samtools v.1.19 (Li et al. 2009). Bedtools v.2.31.1 (Quinlan and Hall 2010) was used for binning aligned reads by alignment position. Coverage visualizations were created with Circos v.0.69.9 (Krzywinski et al. 2009) and Sushi v.1.31 (Phanstiel et al. 2014).

### 2.13 RNA Isolation and RNA Sequencing

Total RNA was isolated from WCL and EV samples using Trizol, and RNA was purified using RNA Clean & Concentrator Kit with DNase treatment (ZymoResearch, R1013). Total RNA was isolated following Trizol procedure and purified using RNA Clean & Concentrator Kit (ZymoResearch, R1013) following the manufacturer's description. The RNA integrity was confirmed using a Bioanalyzer (Agilent). For total RNA‐seq of WCL samples, rRNA was depleted using an Illumina Ribo‐Zero Plus rRNA Depletion Kit (Illumina, #20040526). All cDNA libraries were prepared using the NEBNext Ultra II Directional RNA Library Prep Kit for Illumina (NEB; #E7760). cDNA libraries were sequenced using the Illumina NextSeq1000 system with 100‐nt read length in paired‐read mode. For RT‐PCR amplification of ncRNAs, EV pellets were directly resuspended in 1 mL Trizol after purification and processed as described above. Extracted total RNA was used as a template to perform gene‐specific RT using SuperScript III (ThermoFisher Scientific, 18080093). cDNA was then used as a template for regular PCR amplification using gene‐specific primers and GoTaq (Promega, M7122) with 45× amplification cycles.

### 2.14 RNA Analysis

The data was preprocessed, mapped and counted with Curare v.0.6.0 (Blumenkamp et al. 2024). This workflow used Trim Galore v.0.6.10 (https://www.bioinformatics.babraham.ac. uk/projects/trim_galore/) for quality control and adapter trimming with default settings. Mapping was performed with Bowtie 2 v.2.5.2 in ‘–very‐sensitive’ mode (Langmead and Salzberg 2012). Mapping results in SAM format were converted with samtools v.1.18 (Li et al. 2009). Features (CDS, ncRNA, tRNA and rRNA) were counted with featureCounts v.2.0.6 (Liao et al. 2014) with ‘‐p –countReadPairs ‐s 2’. The genome GCF_000007245.1 and the new reannotation were used for the alignment and feature assignment, respectively.

### 2.15 NcRNA Analysis

Sequences of *sXFs* were retrieved to perform sequence and secondary structure alignments using Copra‐Tool (http://rna.informatik.uni‐freiburg.de; version 5.0.10) (Wright et al. 2013; Wright et al. 2014; Raden et al. 2018).

### 2.16 sRNA Target Prediction in *X. fastidiosa*

To predict targets in *Xff* Tem1, the intaRNA tool (http://rna.informatik.uni‐freiburg.de; version 5.0.10) (Wright et al. 2014; Raden et al. 2018, Mann et al. 2017; Busch et al. 2008) was run locally against the newly annotated *Xff* Temecula1 genome with high‐confidence interactions when *p* value < 0.05.

### 2.17 sRNA Target Prediction in Host Plants

For target prediction in host plants of *Xf*, *sXF* fragments were generated employing a sliding‐window approach. Target prediction was performed using psRNATarget (https://www.zhaolab.org/psRNATarget) (Dai et al. 2011; Dai and Zhao 2011; Dai et al. 2018) using the *Arabidopsis thaliana* Col‐0 Reference genome TAIR 10 (JGI genomic project, Phytozome 13, 447_Araport11) and the *Vitis vinifera* genome PN40024.v4. PsRNATarget uses a mismatch‐sensitive ‘seed’ region (Nucleotides 2–7) and mRNA target accessibility to identify and classify target genes. To increase prediction precision and reduce predicted candidates, we focussed our analysis on targets of 24‐nt fragments. Interactions were classified high‐confidence when *E* value ≤ 3. For overrepresentation (OR) analysis in *Arabidopsis* targets, Panther online tool was used (Panther19; https://pantherdb.org/) (Mi et al. 2019; Thomas et al. 2022). OR on *V. vinifera* targets and *Xf* transcripts were performed with the packages clusterProfiler and enrichplot in R.

### 2.18 Plant Infection Assays

Fourteen‐days‐old sterile‐grown *A. thaliana* Col‐0 seedlings were infected with 5 µL *Xff* Tem1 inoculum (OD_600_ = 0.2 in 1× PBS, pH 7.4) or 5 µL 1× PBS (mock) by pricking with 27G needles. Seedlings were harvested 3 days postinoculation and snap‐frozen until further use.

### 2.19 EV Infiltration Assays

Fourteen‐days‐old sterile‐grown *A. thaliana* Col‐0 seedlings were transferred into 500 µL liquid ½ Murashige and Skoog media overnight. Then, media was removed and seedlings were flooded in either 500 µL 1× PBS pH 7.4 (mock) or 500 µL EVs collected from supernatants of 400 mL *Xff* Tem1 cultures after 0.22 µm filtering, centrifugation at 38,000 × *g* for 1 h to remove cellular debris and then further centrifugation at 150,000 × *g* for 4 h. Vacuum was applied twice for 30 s. Seedlings were harvested 4 h later and snap‐frozen until further use.

### 2.20 Seedling Gene Expression Assays (RT‐qPCRs)

Total RNA of seedlings was extracted using the CTAB‐Method (Bemm et al. 2016) and DNase treated using Turbo DNase (Invitrogen, AM2238). qRT‐PCRs were performed with 10 ng RNA using the NEB Luna Universal One‐Step RT‐qPCR Kit (E3005), in 10 µL reactions according to manufacturer guidelines (55°C 10 min, 95°C 1 min, 95°C 10 s, 60°C 30 s, 45 cycles and subsequent melting curve analysis) using primers according to Data S4. *CDKA* was used as a housekeeping gene, and expression values were analysed using the 2^(−ΔΔct)^ method (Livak and Schmittgen 2001) comparing treated samples (*Xff* Tem1 or *Xff* Tem1‐EVs) to an average of respective mock samples. Significance was assessed by two‐sided Student's *t* test (*α* = 0.05, *p* values *< 0.05, ** < 0.01) using the stats‐package in R.

### 2.21 Genome Annotation

The genome of *Xff* Tem1 (NCBI accession GCF_000007245.1) was reannotated using the Bakta pipeline (Schwengers et al. 2021). The full annotation can be found in the Zenodo repository DOI 10.5281/zenodo.13970767.

### 2.22 Statistical Analysis

All downstream analyses were performed using R version 2024.04.2+764.

## 3 Results

### 3.1 EVs Contain 301 Proteins in *Xff* Tem1 and 140 Proteins in *Xfp* DD

To identify potential effectors secreted by EVs, we determined the proteomes of purified EVs following MISEV guidelines (Lötvall et al. 2014; Théry et al. 2018; Witwer et al. 2021; Welsh et al. 2024). EVs were isolated from bacterial culture filtrates using differential ultracentrifugation followed by SEC (Figure S1a). SEM confirmed the characteristic vesicular structures (Figure S1b), with no apparent contamination from pili, ribosomes or phage particles. The vesicle diameters ranged from 70 to 300 nm, with a median of 136 nm (Figure S1c). The *ζ*‐potential measurement showed an initial charge of −32.5 mV, which became less negative upon Proteinase (Prot)K treatment alone (−8 mV) and cotreatment with MNase (−20 mV) (Figure S1d). This indicates that the EV corona's protein and nucleic acid composition significantly influence vesicle charge. The more negative charge observed in cotreated EVs may be due to cell wall components like LPS (Rapicavoli et al. 2018; Sabra et al. 2003). Enzymatic removal of the EV corona did not affect median particle concentration but changed particle charge (Figure S1c–e), suggesting that the EV corona composition can influence vesicle biophysical parameters.

**FIGURE 1:**
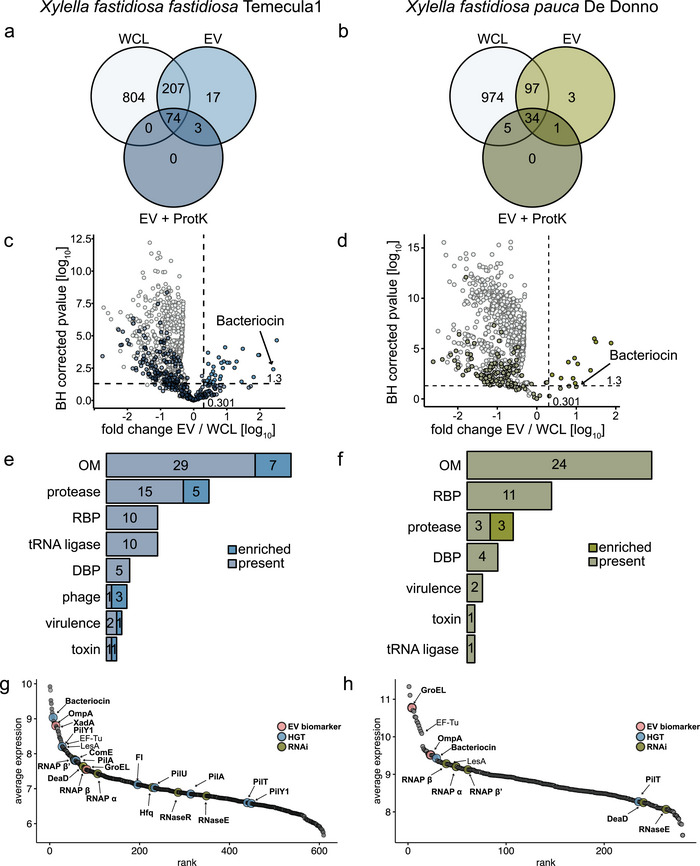
Proteomic analysis identifies 301 and 140 proteins enriched in *Xff* Tem1‐ and *Xfp* DD‐EVs, respectively. Comparison of proteins detected in whole‐cell lysate (WCL), untreated EVs and EVs treated with Proteinase (Prot) K of *Xff* Tem1 (a, c, e, g) and *Xfp* DD (b, d, f, h). (a, b) Proteomic analysis on four biologically independent samples; proteins were considered when identified in *n* ≧ 3 replicates. (c, d) Volcano plots comparing WCL and EVs. EV proteins were categorized as enriched in EVs compared to WCL when fold change EV/WCL > 2 and BH corrected *p* value < 0.05 or present in EVs when identified in *n* ≧ 3 replicates; bacteriocin is highlighted as highly enriched EV protein. (e, f) EV proteins were manually categorized in DBP, RBP, tRNA ligases, virulence, phage, toxins, proteases and outer membrane (OM) proteins depending on their described function and number of identified proteins per category are displayed. (g, h) Rank plot analysis of highly abundant EV proteins; proteins potentially suitable as EV biomarker or proteins involved in horizontal gene transfer (HGT) or RNA interference (RNAi) are highlighted. EV, extracellular vesicle; *Xff*, *X. fastidiosa* subsp*. fastidiosa*; *Xff* Tem1, *Xff* strain Temecula1; *Xfp*, *X. fastidiosa* subsp*. pauca*; *Xfp* DD, *Xfp* strain De Donno.

To determine the protein composition of *Xf*‐EVs, we characterized purified vesicles using liquid chromatography‐tandem mass spectrometry (LC‐MS/MS) and compared it with proteomes from ProtK‐treated EVs and WCL. A total of 1’105 proteins were identified in *Xff* Tem1 and 1’114 in *Xfp* DD across at least three replicates per sample type (Data S1). Principal component analysis (PCA) revealed systematic differences between the proteomes of the two sample types (WCL and EVs), with *Xff* Tem1 showing PC1 at 74.1% and PC2 at 10.5%, and *Xfp* DD showing PC1 at 88.4% and PC2 at 6.8% (Figure S2c, d). We identified 301 proteins in *Xff* Tem1‐EVs, representing 27% of all *Xff* Tem1 proteins, while *Xfp* DD‐EVs contained 140 proteins, approximately 13% of all *Xfp* DD proteins (Figure 1a, b, Data S1). Previous proteomics studies reported 99 and 123 proteins in crude *Xff* Tem1 vesicle samples (Feitosa‐Junior et al. 2019; Nascimento et al. 2016), of which 13.6% and 32% were among the 301 *Xff* Tem1‐EV proteins, respectively (Data S1).

Approximately two‐thirds of the identified vesicle‐associated proteins are sensitive to ProtK treatment, with 74.42% in *Xff* Tem1‐EVs and 71.43% in *Xfp* DD‐EVs (Figure 1a, b). The proteins detected in EVs were classified into two categories: (i) those enriched in EVs compared to WCL and (ii) those present in EVs (Figure 1c, d). Since EV proteomes differ significantly from those of WCL, this supports the existence of a selective mechanism for the delivery of protein cargoes to EVs (Toyofuku et al. 2023; Haurat et al. 2011).

We conducted GO analysis on all EV‐associated proteins to investigate the cellular components (CCs), molecular functions (MFs) and biological processes (BPs) in which these proteins are involved. For *Xff* Tem1‐EVs, 11 significantly enriched GO‐terms were identified, primarily associated with the outer membrane (OM) compartment (for CC), peptidase activity (for MF), proteolysis and protein metabolic processes (for BP) (Figure S3a). In *Xfp*‐EVs, 22 GO‐terms were revealed, including additional terms related to ribosomal proteins (for CC), tRNA binding, rRNA binding, unfolded protein binding, protein folding chaperones and antioxidant activity (for MF). These terms also encompassed responses to toxic substances and oxidative stress, protein maturation and folding, detoxification and responses to chemical stimuli (for BP) (Figure S3b). Based on their functions, we further classified the EV proteins into eight categories: OM proteins, proteases, toxins, phage‐related proteins, virulence factors, tRNA ligases, as well as DNA‐binding proteins (DBPs) and RNA‐binding proteins (RBPs); ribosomal proteins and proteins not fitting in any apparent category were not included (Figure 1e, f).

In both subspecies, the OM class contained the largest number of EV proteins (36 in *Xff* Tem1‐EVs and 24 in *Xfp* DD‐EVs), including TonB‐dependent receptors, porins and fimbrial proteins (Data S1). The detection of OM proteins in our vesicle samples aligns with the hypothesis that *Xf* releases a significant proportion of EVs as OMVs through budding from the OM (Avila‐Calderón et al. 2021). A rank analysis of all EV proteins revealed that EF‐Tu is one of the most highly abundant protein cargoes in EVs for both subspecies (Figure 1g, h), highlighting the immunomodulatory capacity of *Xf* EVs (Mitre et al. 2021). Moreover, proteases were prevalent in EV samples from both *Xff* Tem1 (20 proteins) and *Xfp* DD (six proteins), including the putative lipase/esterase LesA. Given that LesA is crucial for *Xff* Tem1 virulence in grapevine (Nascimento et al. 2016), this suggests that EVs isolated from cultured bacteria may contain proteins pertinent to *Xf* infection. This is further supported by the identification of three *Xff* Tem1‐ and two *Xfp* DD‐annotated virulence proteins in the EV samples (Figure 1e, f, Data S1), including two AcvB‐like proteins (Q87AC4, Q879Z9), VirK‐like family proteins (Q87D31, UPI0003D31AC0) and a nucleoid structuring protein (H‐NS histone family, virulence regulator; UPI00000C274D). Additionally, we identified 11 uncharacterized proteins in *Xff* Tem1‐EVs (Data S1), which match parameters for computational prediction of candidate effectors such as a molecular mass of less than 30 kDa and the absence of transmembrane domains (Dalio et al. 2018).

### 3.2 The Shared *Xf*‐EV Proteome Includes Known EV Markers, DBPs and RBPs

**FIGURE 2:**
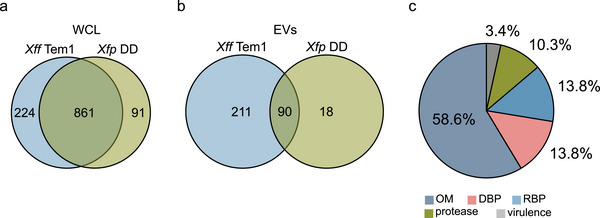
*Xff* Tem1‐ and *Xfp* DD‐EVs share many DNA‐ and RNA‐binding proteins. (a) Overlap of *Xff* Tem1‐EV and *Xfp* DD‐WCL proteins. (b) Overlap of *Xff* Tem1‐EV and *Xfp* DD‐EV proteins. (c) Pie chart representing functional categories of the 90 shared proteins between *Xff* Tem1‐ and *Xfp* DD‐EVs. EV, extracellular vesicle; WCL, whole‐cell lysate; *Xff*, *X. fastidiosa* subsp*. fastidiosa*; *Xff* Tem1, *Xff* strain Temecula1; *Xfp*, *X. fastidiosa* subsp*. pauca*; *Xfp* DD, *Xfp* strain De Donno.

Our next objective was to identify the EV proteome shared by both *Xf* subspecies. Utilizing the annotated reference proteome for *Xf* (*Xff* Tem1), we identified 861 proteins shared between the cellular sample of both subspecies (Figure 2b) and 90 proteins common to both *Xff* Tem1‐ and *Xfp* DD‐EVs (Figure 2b, Data S1). More than half (58.6%) of the *Xf*‐EV shared proteome consisted of proteins associated with the OM (Figure 2b). This includes the chaperonin GroEL (Q87BC0) as well as the OM proteins OmpW and OmpA (Q87AW4), a homologue of OM porin OprF in *Xf* (Table 1). GroEL was the third most abundant protein in *Xfp* DD‐EV samples (Figure 1h). It is commonly found at EVs of various bacterial species and well‐established as an EV biomarker (McCaig et al. 2013; Daleke‐Schermerhorn et al. 2014; Yu et al. 2024). OmpW, abundantly present in OMVs and OprF, involved in OMV biogenesis, are recognized as biomarkers for OMVs from Gram‐negative bacteria (Janda et al. 2023; Mathur et al. 2023; Ojima et al. 2018). The identification of shared EV proteins across *Xff* Tem1 and *Xfp* DD extends the known EV biomarkers for *Xf*, providing additional insights into conserved mechanisms of vesicle biogenesis.

**TABLE 1:** Shared EV proteins of *Xff* Tem1 and *Xfp* DD of category ‘outer membrane’ and known bacteria EV biomarkers found in EVs of both subspecies.

Uniprot	Gene	Locus tag	Description	Rank position in *Xff* Tem1 and *Xfp* DD
Q87AW4	ompA	PD_1924	Outer membrane porin	14/23
Q87BC0	groEL/groL/mopA	PD_1538	Chaperonin GroEL	81/4
Q879×4	cirA	PD_2065	TonB‐dependent receptor	15/15
Q87AR6	bamD	PD_1756	Outer membrane proteiein assembly factor BamD	39/255
Q87C13		PD_1283	TonB‐dependent receptor	7/12
Q87CZ4	pal	PD_0895	Peptidoglycan‐associated protein	37/189
Q87CZ5	tolB	PD_0894	Tol‐Pal system protein TolB	32/84
Q87EI1	oma	PD_0326	Outer membrane protein assembly factor BamA	24/140
Q87EI9		PD_0318	TonB‐dependent receptor‐like beta‐barrel domain‐containing protein	17/69
Q87EN9	oprO	PD_0264	Porin O	27/13
Q87AL6	ompW	PD_1807	Outer membrane protein	1/2
Q87DC8	pcp	PD_0757	Peptidoglycan‐associated outer membrane lipoprotein	6/21

Interestingly, while the cellular proteomes of *Xff* Tem1 and *Xff* DD exhibited a substantial overlap, their EV proteomes were distinctly different (Figure 2a, b). The cellular proteomes revealed an overlap of 861 proteins between the two subspecies, representing 79% of all cellular proteins in *Xff* Tem1 and 90% in *Xfp* DD (Figure 2a, b). Although 83% of the proteins found in *Xfp* DD‐EVs (90/108) were also present in *Xff* Tem1‐EVs, 70% of the proteins in *Xff* Tem1‐EVs (211/301) were unique to this subspecies and absent in *Xfp* DD‐EVs. Notably, we detected nearly three times more proteins in *Xff* Tem1‐EVs compared to *Xfp* DD‐EVs (301 vs. 108, Figure 2b). Given that the protein counts in the WCL samples were similar (1085 for *Xff* Tem1 and 952 for *Xfp* DD, Figure 2a), these findings suggest that each subspecies regulates protein sorting into EVs differently. Notably, we observed strain‐specific differences, as *Xff* Tem1 released more EVs than *Xfp* DD when grown at similar ODs under the same culture conditions (Figure S2a), suggesting that *Xff* Tem1 has a more active vesiculation process.

The shared *Xf*‐EV proteome was enriched for nonribosomal RBPs and DBPs, each comprising 13.8% of the proteome (Figure 2c, Figure 1e, f). Many of these proteins were sensitive to ProtK treatment (Data S1), indicating their likely presence at the vesicle corona. Among the identified RBPs is EF‐Tu, which has been previously identified in crude vesicle samples and is known to contribute to vesicle immunogenicity (Feitosa‐Junior et al. 2019; Mitre et al. 2021, Nascimento et al. 2016). We also found the RNA helicase DeaD and ribonuclease E, known to play roles in RNA metabolism, at EVs from both subspecies (Data S1) (Hussain 2024; Mackie 2013). By contrast, the RNA chaperone Hfq and Ribonuclease G were only detected in *Xff* Tem1‐EVs. Additionally, *Xff* Tem1‐EVs contain 11 tRNA ligases (Arg, Asp, Asn, Cys, His, Ile, Leu, Met, Phe, Thr and Val) (Data S1, Figure 1e), with one (Val) also present in *Xfp* DD‐EVs (Data S1, Figure 1f). Most of these EV‐associated tRNA ligases were sensitive to ProtK treatment, indicating their likely exposure on the vesicle corona.

We identified DBPs in EV samples from both subspecies (Data S1, Figure 1e–g), including DNA‐directed RNA polymerase subunits (Table 2, Figure 1g, h). The DNA transport competence protein ComE (Q87BA0) was detected in *Xff* Tem1‐EVs but not in *Xfp* DD‐EVs (UPI0000007D60). ComE is located in the periplasm and plays a crucial role in facilitating the uptake of external DNA during natural transformation competence, a process well‐documented in Gram‐negative bacteria (Seitz and Blokesch 2013; Johnston et al. 2014). Since natural transformation competence has been described in *Xff* but not in *Xfp* (Kung and Almeida 2011; Kung et al. 2013; D'Attoma et al. 2020), we further explored *Xff* Tem1‐EV proteins potentially involved in HGT. These include key components of the Type‐IV pilus (transformation pilus) consisting of PilA (Q87AA4), PilQ (Q87AX2), PilU/T (Q87CD3/Q87CD4) and PilY1 (Q87E25/Q87FA5). We also identified a phage protein with DNA‐binding function (Q877CH6) (Table 2). Components of the transformation pilus were not identified in *Xfp* DD‐EVs (Data S1, Figure 1e–g).

**TABLE 2:** EV proteins of *Xff* Tem1 with reported roles in HGT.

Uniprot	Gene	Locus tag	Description	Role in HGT	Reference
Transformation pilus/competence proteins					
Q87AA4	pilA	PD_1924	Fimbrial protein	Major pilin subunit of (pseudo‐) pilus structures	(Mackie 2013)
Q87AX2	pilQ	PD_1691	Fimbrial assembly protein	DNA transport across outer membrane	(Shenkutie et al. 2023)
Q87CD3, Q87CD4	pilU/T	PD_1148/PD_1147	Twitching motility protein	Pilus retraction proteins (ATPases), DNA pulling, indispensable for competence	(Moriano‐Gutierrez et al. 2020)
Q87BA0	comE	PD_1558	DNA transport competence protein	DNA import	(Johnston et al. 2014)
Q87E25, Q87FA5	pilY1	PD_0502/PD_0023	Type IV pilus biogenesis factor PilY1 homologue	Pilus assembly, twitching mobility, adhesion to host cell, antiretraction factor, tip of T4P, ‘binds’ to DNA (?)	(Touzdjian Pinheiro Kohlrausch Távora et al. 2022)
Toxin					
Q87BM1	frpC	PD_1427	Bacteriocin, toxin	Increases available DNA‐pool	(Wholey et al. 2016; Kjos et al. 2016)
Other					
Q87CH6	FI	PD_1094	Phage‐related contractile tail sheath protein	DNA packaging protein, structural component of bacteriophage tail (sheath)	

Pilus structures, composed primarily of the major pilin subunit PilA, bind extracellular double‐stranded (ds)DNA and internalize it through the secretion pore PilQ, driven by pilus retraction. This process is powered by retraction ATPase such as PilU and PilT (Q87CD3, Q87CD4), which were also found in *Xff* Tem1‐EVs. Together, these proteins and ComE are essential for natural transformation competence in bacteria (Seitz and Blokesch 2013; Johnston et al. 2014). Thus, we speculate that EVs may play a role in facilitating HGT in *Xff* Tem1, as documented in other bacteria (Tran and Boedicker 2017; Marinacci et al. 2023; Dell'Annunziata et al. 2021).

Interestingly, bacteriocin was one of the most abundant proteins in EVs of both strains of *Xf‐*EVs, with its levels significantly enriched compared to WCL samples (Figure 1c, d, g, h). Bacteriocin has antimicrobial properties, inhibiting the growth of bacterial strains or species (Sugrue et al. 2024). In *Streptococcus pneumoniae*, bacteriocin expression has been linked to genetic adaptation through the predation of neighbouring cells (Wholey et al. 2016). By eliminating or inhibiting competitors, bacteriocin may help *Xf* to establish its niche dominance (Lacava et al. 2004; Deyett et al. 2017; Giampetruzzi et al. 2020; Vergine et al. 2020; Zicca et al. 2020; Anguita‐Maeso et al. 2022).

### 3.3 *Xff* Tem1‐EVs Are Enriched in Genomic Islands (GIs)

**FIGURE 3:**
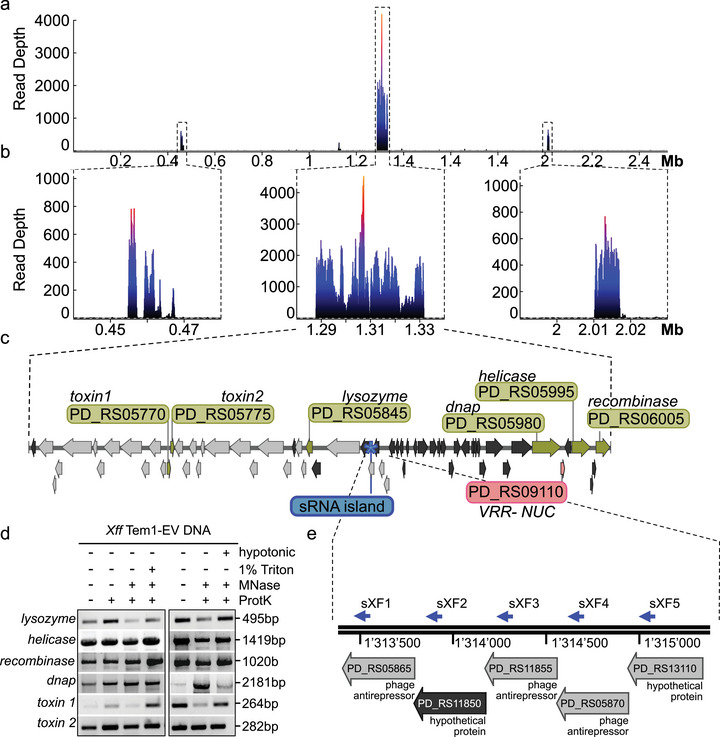
DNA sequencing identifies three genomic islands enriched in *Xff* Tem1‐EVs, including island‐coding *sXF*s. (a, b) Mapping of sequencing reads to the reference chromosome sequence of *Xff* Tem1. Coverage plots show enrichment for three genomic regions: Region 1 from 454,900 to 468,200 bp with a length of 13.3 kb, Region 2 stretches from 1,287,800 to 1,331,700 bp (43.9 kb) and Region 3 from 2,009,200 to 2,017,100 bp (7.9 kb). (c) Region 2 contains many proteins of unknown function (dark grey) and phage proteins (light grey). It also contains genes encoding for two toxins (*toxin1* PD_RS05770; *toxin2* PD_RS05775), *lysozyme* (PD_RS05845), *DNA polymerase* (*DNAP*, PD_RS05980), *VRR‐NUC domain‐containing protein* (*VRR‐NUC*, PD_RS09110), *DEAD/DEAH box helicase* (PD_RS05995) and *recombinase/integrase* (PD_RS06005). Visualization was done using SnapGene software (www.snapgene.com). (d) Genes encoding *toxin1, toxin2, lysozyme*, *helicase*, *recombinase* and *dnap* can be amplified from *Xff* Tem1‐EVs untreated or treated with Proteinase K (ProtK) alone or in cotreatments with MNase, in EVs disrupted by 1% Triton‐X‐100 treatment or burst in hypotonic buffer. Similar results were observed in at least two independent experiments. (e) Region 2 also contains a sRNA‐island of five homologous *sXFs*. EV, extracellular vesicle; *Xff*, *X. fastidiosa* subsp*. fastidiosa*; *Xff* Tem1, *Xff* strain Temecula1.

Due to the higher vesicle production activity of *Xff* Tem1 and the abundance of DBPs with roles in HGT identified in its EVs (Table 2), we focussed our subsequent experiments on this strain to investigate the presence of DNA cargo. DNA‐seq of cellular DNA from *Xff* Tem1 revealed uniform coverage across the entire genome (Figure S4a). By contrast, DNA sequences from purified *Xff* Tem1‐EVs showed enrichment in three specific genomic regions (Figure S4b). Using 100 nt bins and averaging coverage across four replicates, we identified three regions with significantly higher coverage (Figure 3a, b, Figure S4b): Region 1 (454,900–468,200; coverage of 855), Region 2 (1,287,800–1,331,700; coverage of 4701) and Region 3 (2,009,200–2,017,100; coverage of 820). These regions exhibited a higher GC content compared to the genome average (57%, 57% and 58% vs 52%, respectively). The sequences within these regions predominantly encoded phage‐related genes and genes coding for proteins with unknown functions (Figure 3c, Figure S5c, d). Regions 1 and 2 also included genes for the Type‐II toxin‐antitoxin system, DNA polymerase (*DnaP*), a DEAD/DEAH box helicase, a tyrosine‐type recombinase/integrase and various toxins (Figure 3c, Figure S5c, d, Data S2).

Given that Region 2 showed the highest read coverage, we focussed on this region for further analysis. PCR‐based amplification confirmed the presence of genes from Region 2 in *Xff* Tem1‐EVs (Figure 3d). These genes were resistant to treatments with ProtK alone and cotreatment with MNase, suggesting that they are not associated to the ProtK‐sensitive EV corona. Additional cotreatment with 1% TritonX‐100 did not remove these PCR signals (Figure 3d). To support this observation, we burst EVs with hypotonic buffer treatment. Notably, cotreatments with ProtK and MNase also did not eliminate the PCR signals (Figure 3d), indicating that the DNA cargo is not freely dispersed within the vesicle lumen. TEM confirmed that treatment with 1% Triton X‐100 or hypotonic buffer led to the breakdown of EV structures, with hypotonic buffer treatments revealing longish membrane fragments (Figure S6a). It is possible that the GI‐encoded genes are not freely enclosed within the EV lumen but could be directly associated with EV membranes and thereby be protected from MNase digestion.

The high GC content, presence of phage‐related genes, genes encoding tyrosine‐type recombinases/integrases and the insertion near the 3′‐ends of tRNA‐encoding genes suggest that these regions represent GIs (Juhas et al. 2009). Region 2 begins approximately 600 nt downstream of the tRNA‐Arg gene (PD_RS05700) (Figure S7). The presence of GIs in all three regions was confirmed using Atollgen for GI classification (Audrey et al. 2023): Regions 1 and 2 were classified as integrated elements (IEs), containing mobility genes such as ‘Integrase Tyrosine’ with PFAM entries PF13495, PF13102 and PF00589 for phage integrases. Region 2 also includes a Type‐IV mobility gene (*VirB8*) and an endoribonuclease *L‐PSP* (PF01042). Region 3 is an ‘uncharacterized GI’ and includes a Type‐III restriction enzyme (PF04851) and the CRISPR‐associated protein Cse1 (PF09481), suggesting the potential for transferring bacterial immunity islands via this region (Data S2). Of note, all three regions contain genes encoding for VRR‐NUC domain‐containing proteins (Figure 3c, Figure S5). Although little is known regarding the function of this domain in bacteria yet, previous studies proposed an antibacterial effect by inducing double‐strand breaks of genes encoding VRR‐NUC domain‐containing proteins, which could help to enlarge the available gene pool for HGT (Hespanhol et al. 2022).

### 3.4 Genome Annotation Reveals Nine Novel sRNAs Including Island‐Encoding *sXFs*

Since RBPs were present at *Xf*‐EVs (Table 3), we shifted our focus to explore potential RNA‐like effectors, focussing on sRNAs (Wang et al. 2015). First, we inspected the quality reference genome of *Xff* Tem1 (ASM724v1; GCF_000007245.1‐RS_2024_05_05) for the annotation of ncRNAs. Previously, four sRNAs have been annotated: the signal recognition particle sRNA small type‐*ffs* (PD_RS11435), *RNase P* (*RnP*, PD_RS11320), 6S/*SsrS* (PD_RS11670) and the transfer‐messenger (tm)RNA *SsrA* (PD_RS12290) (Table 4). We next reannotated the *Xff* Tem1 genome using Bakta (Schwengers et al. 2021), which includes Infernal versus Rfam ncRNA covariance models, which revealed nine novel sRNAs (Table 4). These sRNAs are homologous to *sX13*, *asX1* and sRNA‐*Xcc1*, which have been linked to virulence in *Xanthomonas campestris* pv. campestris (*Xcc*) and *Xcv* (Schmidtke et al. 2012; Chen et al. 2011). We identified seven homologues of sRNA‐*Xcc1* in the *Xf* Tem1 genome, of which five are encoded by loci that are in close proximity in the genome (1,313,466–1,315,082), clustered within the EV‐associated GI Region 2 (Figure 3e, Figure S7). These five homologues were designated as island‐encoded *sXF1* to *sXF5* (Table 4).

**TABLE 3:** EV proteins of *Xff* Tem1 with potential roles in RNAi.

Uniprot	Gene	Locus tag	Description	Role in RNAi	Reference
RNA binding					
Q87F71	hfq	PD_0066	Hfq	RNA chaperone, sRNA stability, facilitates base pairing sRNA–mRNA	(Santiago‐Frangos and Woodson 2018)
Q87A48/Q87D66	rne/vacB	PD_1983/PD_0820	Ribonuclease E/R	RNA processing/decay	(Mackie 2013)
Q87EU3	deaD	PD_0205	ATP‐dependent RNA helicase DeaD	Unwinding of dsRNA, facilitate protein placement, posttranscriptional regulation	(Iost et al. 2013)
Transcription					
P66713, Q87A32, Q87A33	rpoA/B/C	PD_0461/PD_2001/PD_2000	DNA‐directed RNA polymerase (RNAP) subunits α, β, β′	Transcription of sRNAs/regulation of transcription via sRNAs?	(Wassarman and Storz 2000; Sukhodolets and Garges 2003)

**TABLE 4:** NcRNAs identified in *Xff* Tem1 (ASM724v1) and *Xfp* DeDonno (ASM211787v1) after annotation with Bakta.

Location	Strand	ncRNA	RFAM	RNA‐seq
*Xylella fastidiosa* subsp. *fastidosa* Temecula1				
397,674–397,770	+	Bacterial small signal recognition particle RNA (*SrP*)	RF00169	EV and cell
428,981–429,330	+	Bacterial RNase P class A (*RnP*)	RF00010	EV and cell
483,185–483,185	+	Xanthomonadaceae sRNA *sX13*	RF02232	Cellular only
956,107–956,224	+	Xanthomonadaceae sRNA *asX1*	RF02235	Cellular only
989,110–989,296	—	6S/*SsrS* RNA	RF00013	EV and cell
1,205,173–1,205,258	—	sRNA‐*Xcc*1_1	RF02221	Cellular only
1,215,344–1,215,430	+	sRNA‐*Xcc*1_2	RF02221	Cellular only
1,313,466–1,313,553	—	sRNA‐*Xcc*1_3 (*sXF1*)	RF02221	EV and cell
1,313,852–1,313,937	—	sRNA‐*Xcc*1_4 (*sXF2*)	RF02221	EV and cell
1,314,233–1,314,319	—	sRNA‐*Xcc*1_5 (*sXF3*)	RF02221	EV and cell
1,314,614–1,314,702	—	sRNA‐*Xcc*1_6 (*sXF4*)	RF02221	Cellular only
1,314,998–1,315,082	—	sRNA‐*Xcc*1_7 (*sXF5*)	RF02221	EV and cell
2,447,459–2,447,813	+	Transfer‐messenger RNA, *SsrA*	00013	EV and cell
*Xylella fastidiosa* subsp. *pauca* De Donno				
928,764–928,848	—	sRNA‐*Xcc*1_1	RF02221	nd
949,862–949,978	+	Xanthomonadaceae sRNA *asX1*	RF02235	
998,907–999,092	—	6S / SsrS RNA	RF00013	
1,207,250–1,207,339	—	sRNA‐*Xcc*1_2	RF02221	
1,323,982‐1,324,064	—	C4 antisense RNA	RF01695	
1,324,110–1,324,191	—	*isrK* Hfq binding RNA	RF01394	
1,324,292–1,324,381	—	sRNA‐*Xcc*1_3	RF02221	
1,334,999–1,335,093	+	C4 antisense RNA	RF01695	
1,878,802–1,878,919	—	Xanthomonadaceae sRNA *sX13*	RF02232	
1,918,731–1,919,080	—	Bacterial RNase P class A (*RnP*)	RF00010	
1,950,062–1,950,158	—	Bacterial small signal recognition particle RNA (*SrP*)	RF00169	
2,436,857–2,437,211	+	Transfer‐messenger RNA, *SsrA*	00013	

*Note*: Highlighted in grey are ncRNAs that were previously annotated (GCF_000007245.1‐RS_2024_05_05 and GCA_002117875.1‐ ‐RS_2024_05_05). Highlighted in blue are ncRNAs found in the EV‐packaged GI identified in this paper. RFAM number refers to Database references (rfam.org) and RNA‐seq results indicate if respective ncRNA was identified in cellular samples only or found in *Xff* Tem1‐EVs.

### 3.5 *Xff* Tem1‐EVs Contain 826 Transcripts, Including *sXFs*

**FIGURE 4:**
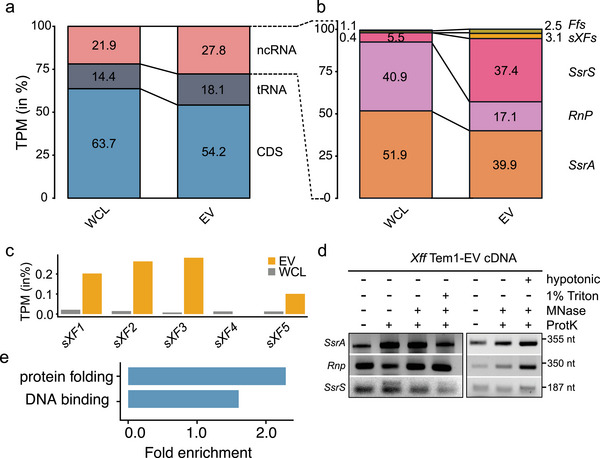
RNA sequencing revealed 826 transcripts in *Xff* Tem1‐EVs including island‐encoded *sXFs*. (a) Proportions of coding‐sequence (CDS), transfer (t)RNA and noncoding (nc) RNA in WCL and *Xff* Tem1‐EV samples. (b) Types of ncRNAs found in WCL and *Xff* Tem1‐EVs are *SsrA* encoding transfer‐messenger (tm)RNA, RNaseP (*RnP*), *SsrS* (or 6S), island‐encoded *sXFs* and signal‐recognition particle (*SrP*, or *ffs*); fractions of ncRNA < 0.1% are not shown (only in WCL: *asX1* – 0.015%; TPP riboswitch – 0.027%; *sX13* – 0.040%. (c) Comparison of TPM of *sXFs* in *Xff* Tem1‐WCL and EVs. (d) Amplification of ncRNAs *SsrA, SsrS* and *Rnp* from cDNA of *Xff* Tem1‐EVs untreated or treated with ProtK alone or cotreated with ProtK and MNase, in EVs disrupted by 1% Triton‐X‐100 treatment or burst in hypotonic buffer. (e) Enriched GO‐terms with adjusted *p* values < 0.05 of *Xff* Tem1‐EV CDS transcripts contain protein folding (GO: 0006457) and DNA binding (GO:0003677). EV, extracellular vesicle; GO, gene ontology; ProtK, Proteinase K; TPM, transcripts per million; WCL, whole‐cell lysate; *Xff*, *X. fastidiosa* subsp*. fastidiosa*; *Xff* Tem1, *Xff* strain Temecula1.

Prompted by the observation that purified *Xff* Tem1‐EVs, labelled with the lipid dye FM4‐64, stained positive for RNA (Figure S8), we further analysed their RNA content by RNA‐seq. We defined EV‐associated transcripts as those present in at least two out of three biological replicates, with an average of at least 10 transcripts per million (TPM). Using this criterion, we identified 826 transcripts in *Xff* Tem1‐EVs and 2071 transcripts in WCL, indicating that approximately 40% of all cellular transcripts are also present in EVs (Data S3). Of the EV transcripts, about half (54%) correspond to coding sequences (CDS), compared to 63.7% in WCL samples (Figure 4a). Notably, there was a higher proportion of tRNAs and ncRNAs in EVs compared to WCL, suggesting selective RNA loading into EVs. This specificity was further supported by the distinct distribution of ncRNA types between EVs and WCL. Although most ncRNAs in WCL samples are *SsrA* and *RnP* (51.9% and 40.9%, respectively), EVs showed a significantly larger proportion of *SsrS* (37.4%), island‐encoded *sXFs* (3.1% compared to 0.4% in cells) and *Ffs* (2.5% compared to 1.1% in cells) (Figure 4b, c).

We confirmed the presence of most abundant ncRNAs at EVs by RT‐PCR, which remained protected from degradation following treatment with ProtK alone or in combination with MNase (Figure 4d). This indicated that *SsrA*, *Rnp* and *SsrS* are not associated to the ProtK‐sensitive EV corona. Combined ProtK, MNase and 1% Triton X‐100 treatment did not eliminate the *SsrA*, *Rnp* and *SsrS* RT‐PCR signals. Given that 1% Triton X‐100 disrupts EV structures and ensuring enzyme activity in these conditions (Figure S6), this suggests that these ncRNAs are not freely enclosed within the EV lumen. Additionally, we burst EVs using hypotonic buffer followed by cotreatments with ProtK and MNase, but the *SsrA*, *Rnp* and *SsrS* RT‐PCR signals persisted (Figure 4d). It is possible that the disruption of EVs by 1% Triton X‐100 or hypotonic conditions generates membrane fragments to which ncRNAs may associate, either directly or indirectly via RBPs like Hfq, which can integrate into membranes (Turbant et al. 2024; Turbant et al. 2023; Mańka et al. 2025). This association could protect the ncRNAs from MNase degradation. Supporting this hypothesis, we consistently observed across independent experiments that the strongest RT‐PCR signal occurred in hypotonic buffer‐burst EVs cotreated with ProtK and MNase (Figure 4d).

To understand the potential functional roles of these vesicular transcripts, we performed GO‐term enrichment analysis on all *Xff* Tem1‐EV‐associated CDS with an average of at least 100 TPM in EVs. This revealed that EV transcripts are enriched for processes such as DNA binding (GO:0003677) and protein folding (GO:0006457) (Figure 4e; Data S3). Notable DNA‐binding transcripts include the phage recombination proteins *Bet* (Q87CQ1) and *FtsK* (Q87DL2), as well as the recombinase *XerD* (Q87DN0). *Xff* Tem1‐EVs also contain transcripts encoding ComE, bacteriocin, four DBPs (H‐NS and HU) and seven DNA‐binding response regulators/transcriptional regulators as well as the transcription termination factor *Rho* and translation initiation factors *IF‐1/‐2/‐3* and two transposases (Data S3). This suggests that EVs may influence DNA structures/chromosome organization and transcription to regulate HGT and stress response mechanisms in *Xf*.

### 3.6 *sXFs* Have 212 Predicted *X. fastidiosa* Targets and Signatures for Hfq Binding

We then focussed on island‐encoded *sXFs* because EVs showed a significantly larger proportion of these ncRNAs compared with cellular ncRNAs (Figure 4c) and exhibited homology to sRNA‐*Xcc1*. In *Xcc*, sRNA‐*Xcc1* is positively regulated by the virulence factors HrpG and HrpX (Chen et al. 2011), which indicates its potential role in bacterial virulence. This involvement might be through (i) regulating the bacterium's gene expression (Wu et al. 2021) or (ii) suppressing host defence mechanisms, as demonstrated in *X*anthomonas *oryzae* pv. *oryzicola* (*Xoc*) (Wu et al. 2024). To investigate this in *Xff* Tem1, we aimed to predict potential mRNA targets of the *sXFs*. We focussed on target predictions within *Xff* as well as in the host plant *V. vinifera* and the genetic model *A. thaliana*, both of which can be colonized by *Xff* (Mitre et al. 2021; Rogers 2012; Pereira et al. 2019).

**FIGURE 5:**
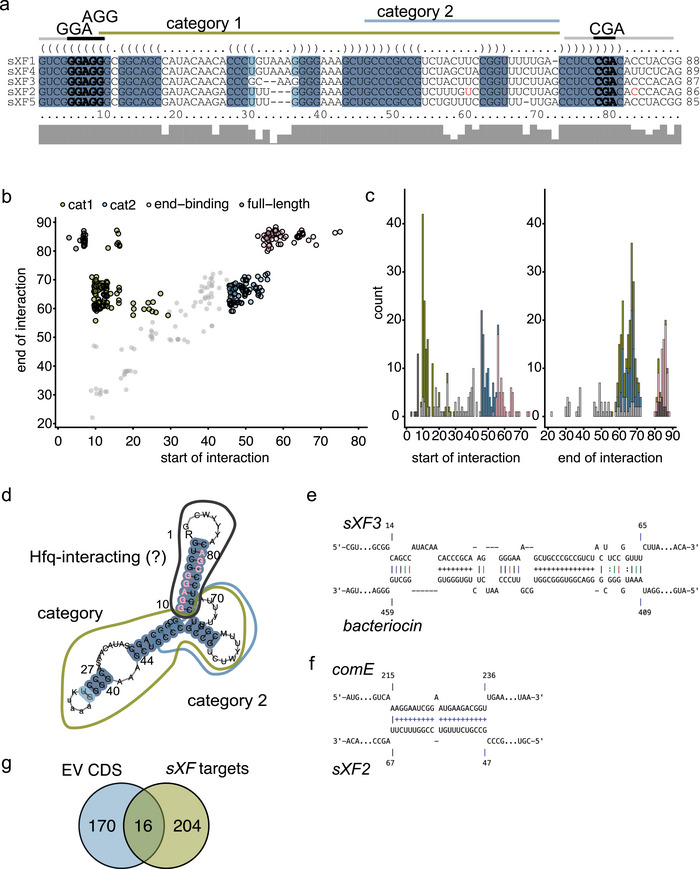
*Xff* Tem1 encodes five sRNA homologues *sXFs*. (a) Five homologues of sRNA‐*Xcc1* show high sequence conservation. (b) Predicted targets of *sXFs* are categorized as full‐length binding (dark grey), end‐binding (pink) or in two categories depending on their start and end of interaction with the target mRNA (Category 1 green, Category 2 blue). Unclassified interactions are depicted in light grey. (c) Most Category 1‐interactions start at position 10 nt of sRNA and most Category 2‐interactions around position 50 nt of the sRNA. (d) Secondary structure prediction of *sXFs*. (e) Interaction of *sXF3* and *bacteriocin* starting at position 14 nt of *sXF3* and ending at position 56 nt. (f) Interaction of *sXF2* and *ComE* starting at position 47 nt of *sXF3* and ending at position 67 nt. (g) Overlap of CDS in *Xff* Tem1‐EVs and predicted *Xff* Tem1 targets of *sXF*s. EV, extracellular vesicle; *Xff*, *X. fastidiosa* subsp*. fastidiosa*; *Xff* Tem1, *Xff* strain Temecula1.

Using IntaRNA (Mann et al. 2017) to predict RNA–RNA interactions between *sXFs*, which are around 90 nt in size (Figure 5a), and *Xff* Tem1 transcripts, we identified 212 potential mRNA targets of *sXFs* in *Xf* (Data S4). IntaRNA takes into account binding energies but also the accessibility of interacting subsequences (Mann et al. 2017). When analysing start and end points of interactions between *sXFs* with their targets, we observed four distinct patterns (Figure 5b): Category 1 includes interactions starting between 9 and 30 nt and ending after 55 nt; Category 2 is interactions starting between 46 and 60 nt and ending before 77 nt; full‐length interactions are lengths greater than 74 nt; and end‐binding starts after 50 nt and ends after 77 nt. We detected a peak in interactions between positions 10 and 20 nt (Category 1) and a second peak around position 50 nt (Category 2) (Figure 5c). For both categories, interactions commonly ended around 60–70 nt of *sXFs* (Figure 5c).

The consensus structure of all *sXFs* revealed three stem‐loop domains (Figure 5d). Given that most interactions occurred between positions 10/50 and 70 nt of *sXFs*, we hypothesized that the first stem‐loop does not directly interact with mRNA targets but may bind RBPs present at the EV corona (Figure 1g). Interestingly, the Hfq binding motif (ARN; Link et al. 2009) is present three times in this stem‐loop (Figure 5a, d). Hfq was shown to interact with single‐stranded RNAs but also binds to folded sRNAs and RNA folds of tRNAs (Link et al. 2009; Vogel and Luisi 2011; Antal et al. 2005). Therefore, we speculate that the ARN‐motif‐containing stem‐loop might be involved in interactions with RBPs such as Hfq. Interactions with mRNAs in Category 1 might involve the remaining stem‐loops, while Category 2 interactions likely involve only the third stem‐loop (Figure 5d). An example for Category 1 is the high‐confidence interaction of *sXF3* with the mRNA encoding bacteriocin (Figure 5e, *p* value < 0.001). The interaction of *sXF2* with the *comE* mRNA is an example for Category 2 (Figure 5f, g, *p* value < 0.01). *comE* is one of the 16 high‐confidence *sXF* targets we identified as transcripts in EVs, following our analysis of the overlap between *sXF* targets and EV‐associated CDS (Figure 5g; Data S4). The presence of both *sXF2* and Hfq in EVs suggests that *comE* transcript levels could be regulated through Hfq‐mediated sRNA‐mRNA pairing, highlighting a potential posttranscriptional control mechanism within *Xff* Tem1‐EVs.

### 3.7 *sXFs* Are Predicted to Target 366 and 632 Genes in Two Different Plant Hosts of *X. fastidiosa*

**FIGURE 6:**
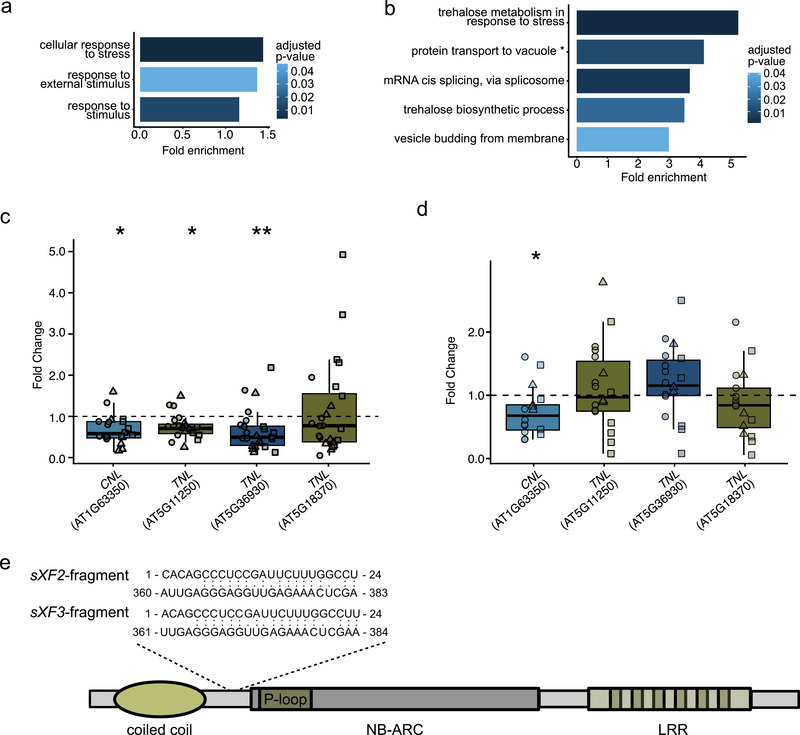
Predicted *sXF*s targets are downregulated during infection and EV treatment. A GO‐term analysis of predicted *in‐planta* targets of *sXF*s are enriched in stress responses in (a) *Arabidopsis thaliana* and (b) the host *Vitis vinifera*. RT‐qPCR results of the predicted target *NLRs* in *A. thaliana* seedlings 3 days postinfection with *Xff* Tem1 (c) and in response to infiltration with *Xff* Tem1‐EVs after 4 h (d) Predicted target site of the *sXF2*/*sXF3* fragment in the *CNL* AT1G63350. *Full‐term: protein transport to vacuole involved in ubiquitin‐dependent protein catabolic process via the multivesicular body sorting pathway. EV, extracellular vesicle; GO, gene ontology; *Xff*, *X. fastidiosa* subsp*. fastidiosa*; *Xff* Tem1, *Xff* strain Temecula1.

To mimic plant miRNA structures, we generated 24 nt‐long fragments of all five homologous *sXFs* using a sliding‐window approach. For target prediction in plants, we utilized ‘seed’‐sensitive psRNATarget (Dai et al. 2018), as previously described (Ren et al. 2019). This analysis initially predicted 2'858 targets in *A. thaliana* and 5'421 targets in *V. vinifera* (Data S4). GO‐term enrichment analysis of the predicted targets revealed: (i) In *A. thaliana*, enriched MF‐terms included responses to external stimuli and stress; and (ii) in *V. vinifera*, enriched MF‐terms included trehalose metabolism in response to stress, mRNA *cis*‐splicing and vesicle budding from membranes (Figure 6a, b).

Applying the same quality cutoff as in previous studies (Ren et al. 2019), we identified 366 high‐confidence targets in *A. thaliana* and 632 in *V. vinifera* (Data S4). Notable high‐confidence targets include: (i) In *A. thaliana*, four disease resistance proteins of the nucleotide‐binding site (NBS) leucine‐rich repeat (LRR) receptor (NLR) class (AT1G63350, AT5G11250, AT5G36930 and AT5G18370); and (ii) in *V. vinifera*, a putative LRR receptor kinase (Vitvi04g01426_t001) and the stress response factor *NAC Secondary Wall Thickening Promoting Factor 1* (*NST1*, Vitvi05g01129_t001), which is a putative target of an *sXF3* fragment (Table 5). NST1 has been associated with lignin biosynthesis and is regulated by xylem‐specific transcription factors in *Arabidopsis* (Zhang et al. 2020; Liu et al. 2021). Increased lignification in the vasculature is a known response mechanism of host plants to *Xf* infections (Sabella et al. 2018). Several other transcription factors are potential targets of *sXFs*, indicating that these fragments may interfere with the regulation of immune responses (Data S4). Interestingly, the transcript of the lipid raft‐associated protein Remorin (Vitvi15g01160_t001) is a putative target of an *sXF1* fragment (Table 5). The association of bacterial EVs with plant membranes involves Remorins, as shown for *Xcc*‐EVs in *A. thaliana* (Tran et al. 2022).

**TABLE 5:** Predicted plant targets of *sXF* fragments using psRNA‐target.

Host	Target plant mRNA	sXF fragment	*E* value	Targeted subdomain	Interaction
*A. thaliana*	*CNL* (AT1G63350)	*sXF2*	3.0	Upstream of P‐loop	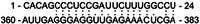
		*sXF3*	3.0		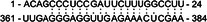
	*TNL* (AT5G11250)	*sXF2*	2.5	Intron	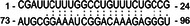
	*TNL* (AT5G36930)	*sXF1*	2.5	TIR‐domain	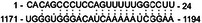
		*sXF5*	2.5		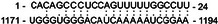
	*TNL* (AT5G18370)	*sXF2*	3.0	Downstream of LRR 9	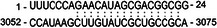
		*sXF5*	3.0		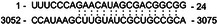
*V. vinifera*	*NST1* (Vitvi05g01129_t001)	*sXF3*	3.0	No subdomain info	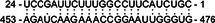
	*Remorin* (Vitvi15g01160_t001)	*sXF1*	3.0	C‐terminal	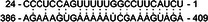

Among the four predicted *sXF*‐targeted *NLRs*, three were downregulated in response to infection with *Xff* Tem1 in *A. thaliana* seedlings (Figure 6c). Downregulation of the coiled‐coil‐domain *NLR* (*CNL*) AT1G63350 was observed in seedlings following exogenous application of *Xff* Tem1‐EVs, whereas the other three tested TIR‐domain *NLRs* (*TNLs*) showed no such response (Figure 6d). Notably, the target site of the *CNL* AT1G63350 is located near the P‐loop domain of the protein, unlike the three *TNLs* (Table 5, Figure 6e). The P‐loop is a conserved sequence targeted by endogenous plant miRNAs from the miRNA482/2118 superfamily to regulate *NLR* expression (Zhang et al. 2022). These findings provide first insights that *Xf*‐EVs could be involved in the interference with immune gene expression.

## 4 Discussion

### 4.1 sRNA Delivery

The success of *Xf* infection has been linked to its ability to produce EVs, which facilitate detachment from surfaces and likely contribute to the delivery of cell wall‐degrading enzymes, promoting systemic infection (Ionescu et al. 2014; Nascimento et al. 2016). Consistent with these roles and previous studies (Feitosa‐Junior et al. 2019; Nascimento et al. 2016), we identified adhesins and the cell wall–degrading enzyme LesA in *Xf*‐EVs (Figure 1g, h). Yet, EVs serve a broader function, as several bacterial pathogens use them to modulate plant immunity in a contact‐independent manner. For example, EVs from the plant pathogens *P. syringae* pv. tomato (*Pto*) DC3000 and *Xcc* carry proteins related to plant immune activation as well as bacterial virulence and stress tolerance adaptation to the plant environment (Janda et al. 2023; Tran et al. 2022; McMillan et al. 2021; Bahar et al. 2016; Chalupowicz et al. 2023). Human bacterial pathogens also use EVs to secrete effectors, as seen in *Salmonella enterica* serovar Typhimurium, whose EVs contain virulence effectors that are encoded in a pathogenicity island (Kim et al. 2018).

EVs from *Xf* have been reported to carry the immune‐activating protein EF‐Tu (Mitre et al. 2021), yet it remained unknown whether they also contain effector‐like molecules. While we identified eleven uncharacterized proteins in *Xff* Tem1‐EVs that meet the criteria for bacterial effector candidates (Data S1; Dalio et al. 2018), this study focussed on the nucleic acid cargo of EVs, as our proteomics analysis revealed DBPs and RBPs at the *Xf*‐EV corona (Figure 2c). Additionally, previous studies have reported sRNAs as effector‐like molecules; for instance, genetic deletion of *sX13* reduced virulence in *Xcv* (Schmidtke et al. 2013), while overexpression of sRNAs in *Xcc* identified RsmU as a negative regulator of virulence and the hypersensitive response (Tang et al. 2020).

In this study, we identified the presence of major RNA types at *Xff* Tem1‐EVs and revealed that approximately 30% of all EV‐associated transcripts in *Xff* Tem1 encode ncRNAs, with proportions differing from those in WCL samples (Figure 4a, b). Similar enrichment of distinct ncRNAs in EVs has been reported for *X. oryzae*, *Pseudomonas aeruginosa*, *Vibrio fisheri* and *Escherichia coli* (Koeppen et al. 2016; Wu et al. 2024; Ghosal et al. 2015; Moriano‐Gutierrez et al. 2020). Although transcripts for all seven homologues of sRNA*‐Xcc1* were detected in WCL samples, the non‐island encoded homologues were absent in EVs. By contrast, *Xff* Tem1‐EVs contained transcripts for four out of the five island‐encoded *sXFs* (Figure 4c). Homologues of *Xff* Tem1 *sXFs* are also present in the *Xfp* DD genome (Table 4); however, they are not clustered and are not encoded within a GI. Moreover, Hfq was detected exclusively in *Xff* Tem1‐EVs and not in *Xfp* DD‐EVs (Figure 1g, h). This suggests that *Xff* Tem1 utilizes EVs to deliver *sXFs* for Hfq‐mediated RNA interference (RNAi), whereas *Xfp* DD may employ other EV‐associated RBPs for RNAi.

The four EV‐associated, island‐encoded *sXFs* are homologous to sRNA‐*Xcc1* (Figure 5a). In *Xanthomonas*, sRNA‐*Xcc1* expression is controlled by HrpG and HrpX, key regulators of the Type‐III secretion system (Teper et al. 2021). This suggests a role of sRNA‐*Xcc1* in virulence, which could involve its transfer into plant cells and host immune modulation similar to the sRNA *Xosr001* from *Xoc* that targets *OsJMT1* in jasmonic acid signalling (Wu et al. 2024). To this end, the predicted high‐confidence plant targets of *sXFs* include four *NLRs*, an *LRR* receptor kinase and *NST1* (Table 5), of which the *CNL* AT1G63350 and the two *TNLs* AT5G11250 and AT5G36930 were downregulated in seedlings infected with *Xff* Tem1 (Figure 6c).

Given that fragments of *sXF2* and *sXF3* are predicted to target an upstream region of the P‐loop in the NB‐ARC domain (Figure 6e, Table 5), a region of *CNLs* known to be targeted by (micro) miRNAs (Zhang et al. 2022; Shivaprasad et al. 2012), we propose that the phasiRNA pathway could integrate longer bacterial ncRNAs, such as *sXFs*, into the eukaryotic RNAi system. Thus, as an extension of cross‐kingdom RNAi, bacterial sRNAs may engage with the phasiRNA pathway, allowing long bacterial sRNAs to be adapted for eukaryotic RNAi. This process could enhance signal amplification and facilitate the silencing of host targets.

ARGONAUTE (AGO) proteins play a key role in the phasiRNA pathway in plants, with AGO1 associating with endogenous sRNAs (Liu et al. 2020). AGO1 can also be hijacked by exogenous sRNAs from both pathogenic and mutualistic microbes, allowing them to suppress host defence genes and promote colonization sRNAs (Liu et al. 2021; Tran et al. 2022). *Bradyrhizobium japonicum* tRNA‐derived sRNA fragments regulate soya bean genes associated with nodule symbiosis through AGO1 (Ren et al. 2019). We showed recently that AGO1 functions as a positive regulator of immunity against *Xff* Tem1 in leaf petioles from mature *A. thaliana* plants (Ruf et al. 2024), suggesting a role of the RNAi pathway in the interaction with *Xf*. Further investigation beyond the scope of this study is required to address how, when and where *sXFs* integrate into this pathway and thereby regulate host immune responses.

The observed downregulation of the *CNL* AT1G63350 in EV‐treated seedlings suggests that *sXFs* may be delivered as virulence factors via EVs. RNA fold analysis predicted that the *sXFs* adopt a stable secondary structure with three stem‐loop structures (Figure 5d). One stem‐loop of *sXF* carries motifs for binding Hfq (Figure 5a, d), which is present at the corona of *Xff* Tem1‐EVs (Figure 1g, Data S1). Hfq is inserted into EV membranes, potentially facilitating the interaction of sRNAs to EV membranes (Turbant et al. 2023). This is consistent with our observation that ncRNA cargo could associate with EV membranes (Figure 4d). Following the observation that *Xcc*‐EVs interact with the plant cell plasma membrane (Tran et al. 2022), a similar scenario could be proposed for *Xf*‐EVs, where ncRNA cargo, due to their association with the EV membrane, may also interact with plant plasma membranes.

Immunomodulation by EV‐sRNAs has been reported across diverse bacteria–host interactions. EVs from *Xoc* contain sRNA Xosr001, which impairs stomatal immunity in rice by regulating OsJMT1 mRNA (Wu et al. 2024). Similarly, *P. aeruginosa* EVs carry sRNA52320 (a methionine tRNA fragment) along with sRNA4518698, sRNA2316613 and sRNA809738, all of which suppress host immune responses (Koeppen et al. 2016; Xie et al. 2024). In *Helicobacter pylori*, EV‐enriched sR‐2509025 and sR‐989262 reduce LPS‐triggered responses (Li et al. 2022). *Flavobacterium psychrophilum* EV‐sRNAs interact with trout immune genes, promoting bacterial coldwater disease (Chapagain et al. 2024). In *E. coli*, the tRNA‐derived fragment Ile‐tRF‐5X is released via EVs and transferred to human cells, where it enhances symbiosis by inducing MAP3K4 expression (Li et al. 2022).

The functions of many sRNAs require Hfq, which binds and stabilizes sRNAs and mediates the base‐pairing with target mRNAs of host cells, leading to repression of translation or acceleration of mRNA decay (Chapagain et al. 2024; Diallo et al. 2022). Hfq has been described as a global posttranscriptional regulator, required, for example, for virulence, colonization, biofilm formation and QS (Waters and Storz 2009). In *Erwinia amylovora*, Hfq and two Hfq‐regulated sRNAs, RprA and RyhA, are important for full virulence of this bacterial pathogen in pear and apple (Zeng et al. 2013). Further knowledge of Hfq‐mediated RNAi could facilitate the development of RNA‐based therapeutic approaches against *Xf*, such as exploiting Hfq for RNA‐based antibacterial gene silencing (Good and Stach 2011).

### 4.2 Vesiduction

sRNAs are also key regulators of various bacterial physiological processes, including stress response, metabolism, motility, QS and virulence gene regulation (Zhang et al. 2022; McMillan et al. 2021). In *Xcc*, RsmU regulates virulence and cell motility by antagonizing RsmA (Ren et al. 2019). Interestingly, possible competence regulatory elements in *Xff* Tem1 were predicted as *sXF* targets (Figure 5f). These included mRNAs coding for the competence proteins ComA and ComE, both involved in DNA binding and uptake (Johnston et al. 2014), the single‐stranded (ss)DBP *SsbB* mRNA and mRNAs coding for Type‐IV PilY/PilW/PlV proteins (Data S4). Generally, the Type‐IV pilus interacts with DNA, conveying it to ComEA that binds dsDNA (Seitz and Blokesch 2013; Johnston et al. 2014). The captured dsDNA is converted into ssDNA and taken up across the membrane by ComEC (Johnston et al. 2014). The entering ssDNA is bound by SsbB and DrpA to integrate into the chromosome (Johnston et al. 2014).

Natural transformation occurs in *Xff* and has been observed under conditions of its natural growth environment of liquid flow (Kandel et al. 2016). Our data suggest that EV‐associated *sXFs* are regulators of natural transformation. Neither ComE nor transformation pili components (Table 2) were detected in *Xfp* DD‐EVs, whereas both are present in *Xff* Tem1‐EVs. Interestingly, only the five island‐encoded *sXFs* are absent from the *Xfp* DD genome, while the other four *sXFs* are present across its genome (Table 4). Taken together, this suggests that *Xff* Tem1‐EVs may function as vesiduction agents, facilitating and regulating natural DNA uptake, a process observed in *Xff* strains but not in *Xfp* strains (Haurat et al. 2011; Avila‐Calderón et al. 2021; Dalio et al. 2018; Chalupowicz et al. 2023; Kim et al. 2018).

Growing evidence suggests that horizontally acquired genes play a major role in shaping the genetic diversity of *Xf* and its virulence, with natural transformation likely being a key mechanism of HGT (Haurat et al. 2011; Schmidtke et al. 2013; Tang et al. 2020, Firrao et al. 2021; Jimenez et al. null). The DNA from the GI carrying the island coding for the five homologues *sXFs*, along with DNA from two other GIs, was enriched in the EVs (Figure 3a, b, Figures S4, S5). GIs are regions of bacterial genomes that are acquired through HGT (Fröhlich and Papenfort 2016). Their interaction with EV membranes suggests protection from adverse environments (Figure 3d), for example, during host infection, and the delivery of the DNA cargo upon association with the OM of surrounding recipient bacterial cells (Wen and Herman 2024), indicative of vesiduction (Soler and Forterre 2020). Studies have also demonstrated the importance of sRNAs in maintaining GIs within bacterial populations. A toxin–antitoxin system encoded by the multidrug resistance *Salmonella* Genomic Island 1 (SGI1) plays a critical role in the stable maintenance of SGI1 in the host chromosome (Huguet et al. 2016). This highlights an intricate relationship between sRNAs and GI in bacterial pathogens.

GIs can regulate biofilm formation and motility in bacteria, as shown in *Vibrio alginolyticus* (Cai et al. 2023). Biofilms are a form of cooperative, sessile lifestyle in bacteria and a persistent pathogenetic mechanisms of chronic infections (Muhammad et al. 2020). The ability to switch between planktonic and biofilm lifestyles is critical for *Xf*’s infection process and correlates with vesiculation (De La Fuente et al. 2022; Ionescu et al. 2014). QS regulates the release of EVs, which results in the detachment of *Xf* from surfaces and switch to planktonic life (Ionescu et al. 2014). Whether the regulation of biofilm formation in *Xf* involves the EV‐enriched genomic regions or the island‐encoded *sXFs*, as shown for various sRNAs in several bacteria (Cai et al. 2023; Van Puyvelde et al. 2013; Sass et al. 2017; Xiao et al. 2017; Shenkutie et al. 2023), remains to be addressed.

**FIGURE 7:**
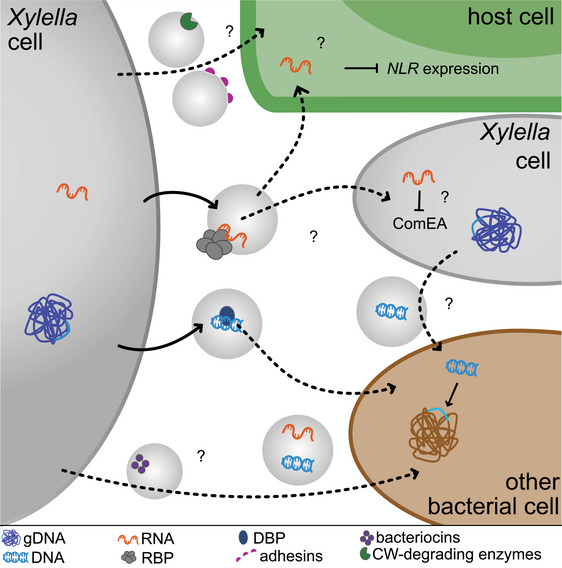
Working model of how *Xff* Tem1uses EVs as a Type‐0 secretion system in a contact‐independent manner. A bacterial donor cell (left) releases EVs with different functional types, illustrated with different cargoes, in response to its physiological state. EVs containing nucleic acids are associated with either RNA or DNA and are decorated with RBPs and DBPs, respectively. The bacterium may utilize EVs for the delivery of *sXFs* and other ncRNAs to recipient host (plant) and bacterial cells, modulating, for example, plant immunity to aid infection, and natural competence in *Xff* Tem1 by influencing HGT. The secretion of GIs including the *sXFs*‐encoding island in EVs as DNA suggests the transfer of this genetic material across the bacterial community and thereby its adaptability to the environment. Other functional types of EVs could be involved in the release of bacteriocin, potentially targeting other bacterial species to dominate the ecological niche as well as cell wall degrading enzymes for systemic plant infection and adhesins to promote the cell's detachment from surfaces. DBP, DNA‐binding protein; EV, extracellular vesicle; GI, genomic island; HGT, horizontal gene transfer; RBP, RNA‐binding protein; *Xff*, *X. fastidiosa* subsp*. fastidiosa*; *Xff* Tem1, *Xff* strain Temecula1.

Our findings propose a model in which *Xf* cells release EVs with at least two functions (Figure 7): One functional type contains RBPs along with ncRNAs such as *sXFs* and *SsrA*, which are intended for delivery to recipient cells. These recipient cells could be plant cells, where they downregulate host immunity genes, or other *Xf* cells, where they influence natural transformation capabilities. This is consistent with the finding that EV‐packaged *SsrA* from *V. fischeri* regulates bacterial activity and contributes to maintaining homeostasis with its squid host *Euprymna scolopes* (Moriano‐Gutierrez et al. 2020). The other functional type of EV contains DBPs and GIs, facilitating HGT of genetic material within the bacterial population. Consequently, the exchange of *sXFs* among bacterial cells could enable widespread regulation of host immunity and natural competence. Other functional types of EVs could deliver cell wall‐degrading enzymes such as LesA and adhesins to regulate surface detachment (Ionescu et al. 2014; Nascimento et al. 2016).

Our data show that *Xff* Tem1 utilizes EVs as a Type‐0 secretion system, enabling vesiduction and the delivery of sRNA to recipient cells without direct physical interaction. This contact‐independent mechanism allows for the potential transmission of genetic information and effectors over longer distances. The absence of the EV‐associated GI and island‐encoded *sXFs* from the *Xfp* DD genome, along with the lack of Hfq and ComE in *Xfp* DD‐EVs, suggests a distinct role for *Xfp* DD‐EVs. Taken together, our proteomic analysis of *Xf*‐EVs presents a putative list of EV markers and suggests strain‐specific differences in their roles in RNAi and HGT. Additionally, our comprehensive data on *Xff* Tem1‐EVs offer an essential molecular framework for understanding the virulence strategies of *Xf* and highlight potential target genes that could be engineered to evade targeting, thereby enhancing plant immunity (Touzdjian Pinheiro Kohlrausch Távora et al. 2022).

## Author Contributions

**Alessa Ruf**: conceptualization (equal), data curation (equal), investigation (equal), methodology (equal), resources (equal), validation (equal), visualization (equal), writing – original draft (equal), writing – review and editing (equal). **Patrick Blumenkamp**: data curation (equal), investigation (equal), methodology (equal), resources (equal), software (equal), writing – review and editing (equal). **Christina Ludwig**: conceptualization (equal), data curation (equal), funding acquisition (equal), investigation (equal), methodology (equal), resources (equal), writing – review and editing (equal). **Anne Lippegaus**: investigation (equal), methodology (equal), writing – review and editing (equal). **Andreas Brachmann**: investigation (equal), writing – review and editing (equal). **Andreas Klingl**: investigation (equal), writing – review and editing (equal). **Alexander Goesmann**: funding acquisition (equal), supervision (equal), writing – review and editing (equal). **Karina Brinkrolf**: supervision (equal), writing – review and editing (equal). **Kai Papenfort**: funding acquisition (equal), investigation (equal), methodology (equal), supervision (equal), writing – review and editing (equal). **Silke Robatzek**: conceptualization (equal), funding acquisition (equal), project administration (equal), supervision (equal), writing – original draft (equal), writing – original draft (equal), writing – review and editing (equal), writing – review and editing (equal).

## Conflicts of Interest

The authors declare no conflicts of interest.

## Supporting information




**Supporting Information Figures**: S1–S8

**Supporting Information Data S1**: (a, b) List of identified proteins in EVs of *Xff* Tem1 and *Xfp* DD, (c) core EV proteins, c, (d) GO enrichments of *Xff* Tem1‐ and *Xfp* DD‐EV proteins.

**Supporting Information Data S2**: (a) Annotation of genes and (b) GI classification of Regions 1–3 identified in DNA‐seq, (c) primers used to amplify genes of Region 2.

**Supporting Information Data S3**: (a) TPM of RNA‐seq, (b) GO enrichments of EV CDS, (c) RT primers used for amplification of ncRNAs.

**Supporting Information Data S4**: (a) Target prediction of *sXFs* in *Xff* Tem1, (b) overlap of EV CDS and *Xff* Tem1 *sXFs*‐targets, (c) target prediction of *sXFs* in *At* and (d) *Vv*, GO enrichments of predicted targets (e, f), (g) qRT‐PCR primers used for gene expression study.

**Supplementary Material**: jev270102‐sup‐0006‐Sourcedata.xlsx

## Data Availability

The mass spectrometric raw files as well as the MaxQuant output files have been deposited to the ProteomeXchange Consortium via the PRIDE partner repository and can be accessed using the identifier PXD056167. The raw data of sequencing can be accessed via EMBL‐EBI (https://www.ebi.ac.uk/) with access code E‐MTAB‐14493 (DNAseq) and E‐MTAB‐14502 (RNAseq). Genome Annotation *Xff* Temecula1 (NCBI accession GCF_000007245.1) with Bakta has been deposited to Zenodo with DOI 10.5281/zenodo.13970767.
