# Supplementary material for: Extracellular Vesicles From Xylella fastidiosa Carry sRNAs and Genomic Islands, Suggesting Roles in Recipient Cells
Source: J Extracell Vesicles. 2025 Jun 25;14(6):e70102. doi: 10.1002/jev2.70102 (PMC12189769; doi:10.1002/jev2.70102)
Supplement: Supplementary file 1 — Supporting Information Figures: S1–S8 [file JEV2-14-e70102-s003.pdf]

Supplemental Figures S1-S9

Dual function of *Xylella fastidiosa* extracellular vesicles in releasing sRNAs and genomic islands for vesiduction

Alessa Ruf[1], Patrick Blumenkamp[2], Christina Ludwig[3], Anne Lippegaus[4], Andreas Brachmann[1], Andreas Klingl[1], Alexander Goesmann[2], Karina Brinkrolf[2], Kai Papenfort[4,5], Silke Robatzek[1, #]

[1]LMU Munich Biocenter, Ludwig-Maximilian-University of Munich, Großhaderner Strasse 4, 82152 Martinsried, DE

[2]Bioinformatics and Systems Biology, Justus Liebig University Giessen, Ludwigsplatz 13-15, 35390 Giessen, DE

[3]Bavarian Center for Biomolecular Mass Spectrometry (BayBioMS), TUM School of Life Sciences, Gregor-Mendel-Strasse 4, 85354 Freising, DE

[4]Bioinstrumentezentrum Friedrich- Schiller University, Institute of Microbiology, Winzerlaer Strasse 2, 07745 Jena, DE

[5]Microverse Cluster, Friedrich Schiller University, Neugasse 23, 07743 Jena, DE

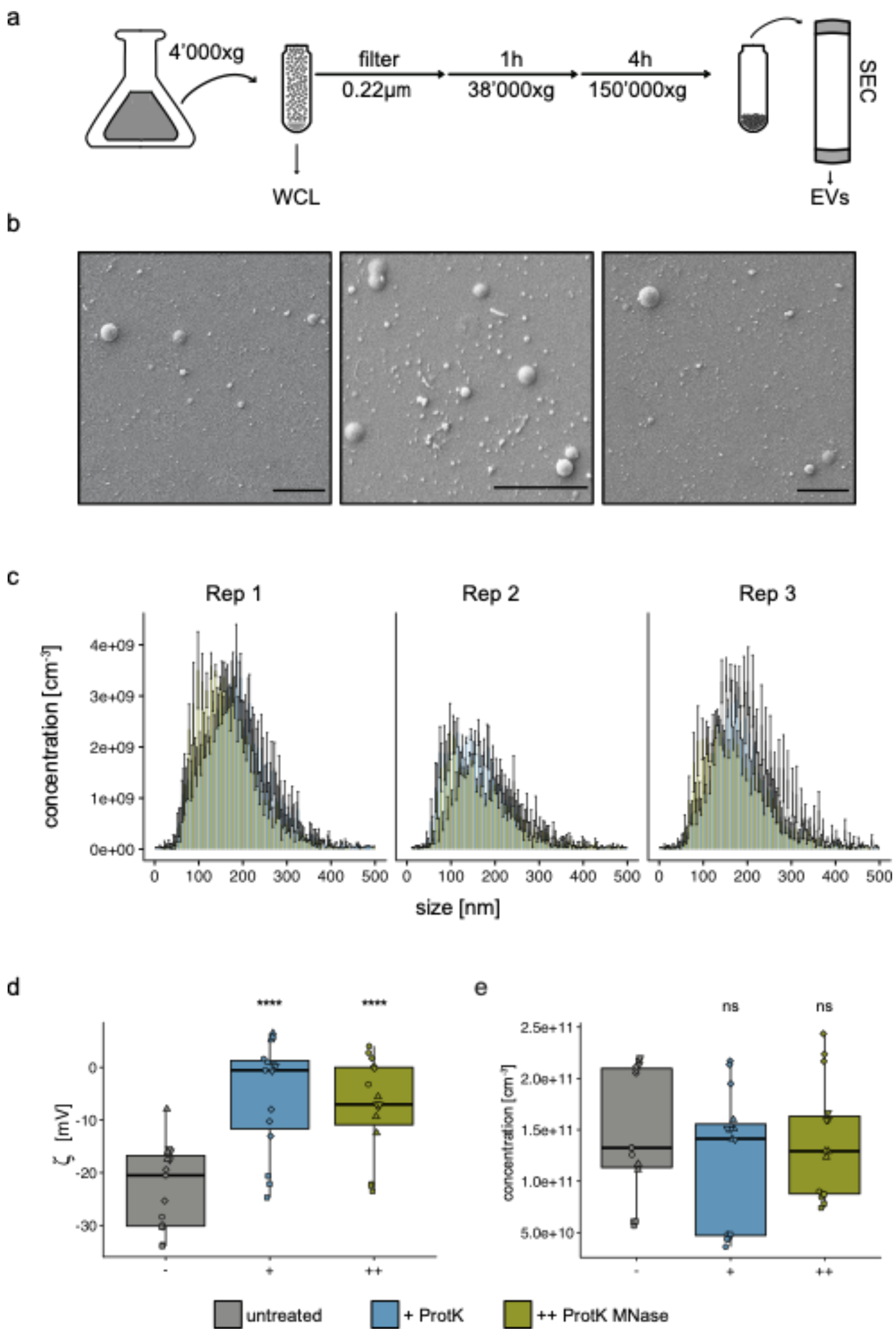

S1: Workflow and biophysical characterization of *Xf*-EVs.
a) Workflow to isolate EVs from axenic cultures using differential ultracentrifugation and size-exclusion chromatography (SEC). **b)** Scanning Electron Microscopy on sucrose-gradient purified OMVs shows typical vesicle-like structures, scale bar 1 µm. **c)** Biophysical characterization of SEC-purified EVs using Nanoparticle-Tracking Analysis (NTA) shows typical size distribution of ca. 70-300 nm sized particles, particles larger as 0.22 µm could represent aggregates. Results of three independent replicates are shown with three measurements each. **d, e)** Enzyme treatment with Proteinase K (ProtK) alone (+) or Proteinase K and MNase (++) did not change overall concentration of particles but their surface charge (Zetapotential ζ); Students t-test with Benjamini-Hochberg adjusted p values = $3.85 \cdot 10^{-5}$). Different symbols show independent replicates.

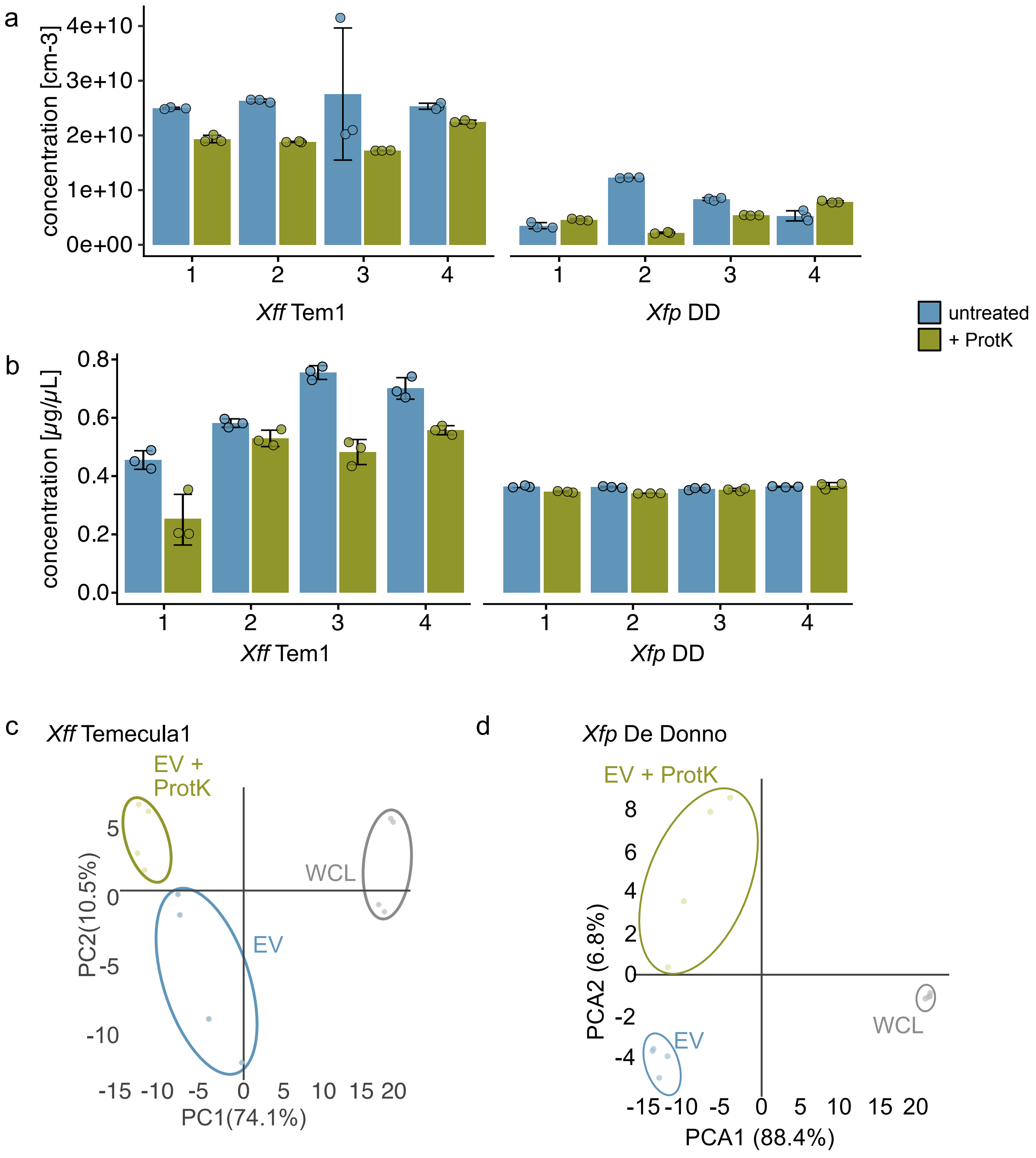

S2: Comparison of untreated and Proteinase K-treated EVs from *Xff* Tem1 and *Xfp* DD cultures for proteomics analysis. a) Biophysical characterization of SEC-purified EVs via NTA shows higher concentration of EVs produced by *Xff* Tem1 compared to *Xfp* DD in same culture conditions and OD_{600} of 0.2 for all samples. The results of three measurements for four independent replicates are shown for each subspecies. **b)** Bradford measurements of EVs show the same trend. **c, d)** PCA of proteomic analysis for *Xff* Tem1 (c) and *Xfp* DD.

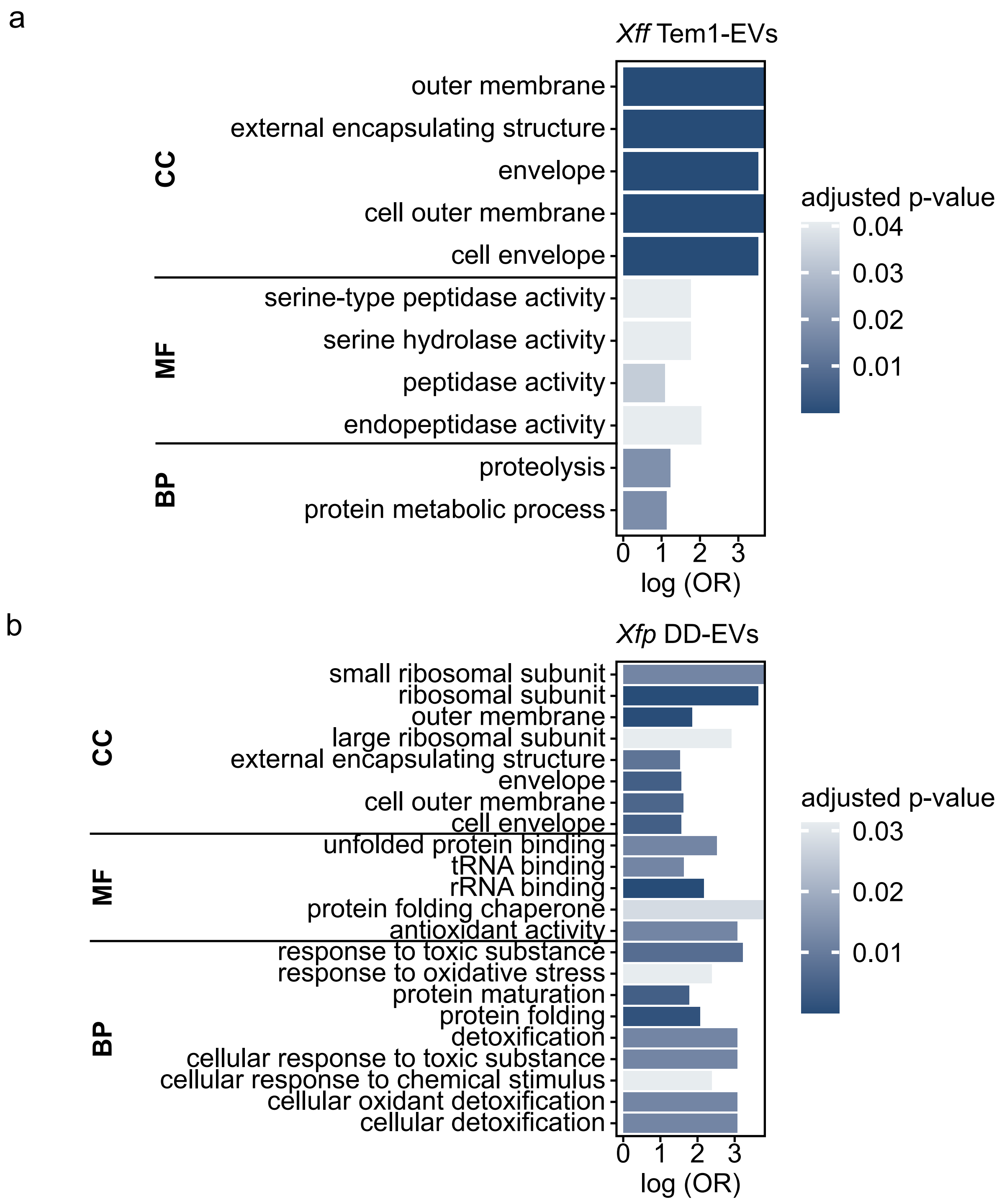

S3: GO enrichment analysis of EV proteins.

a

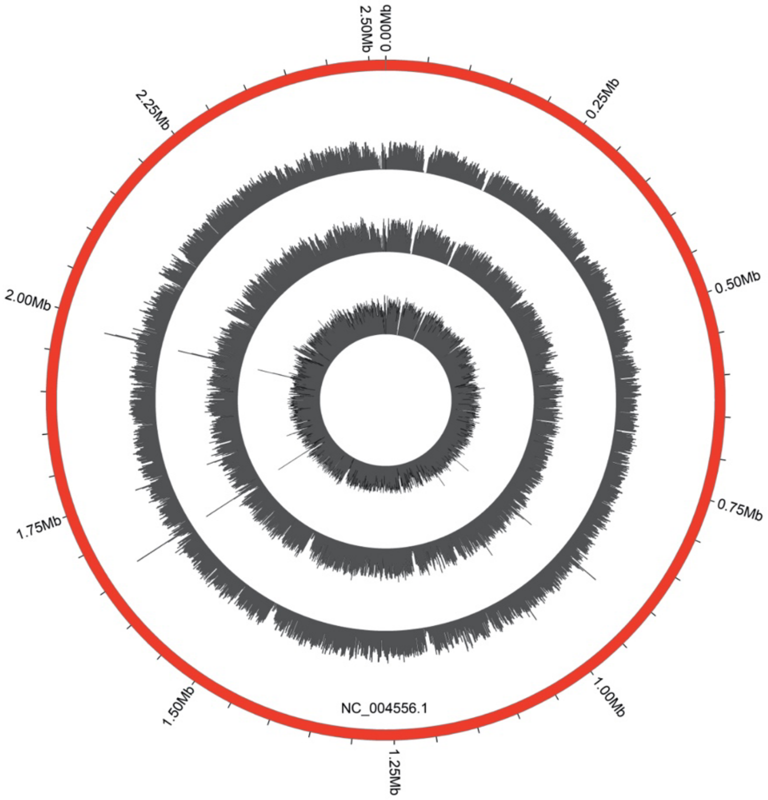

b

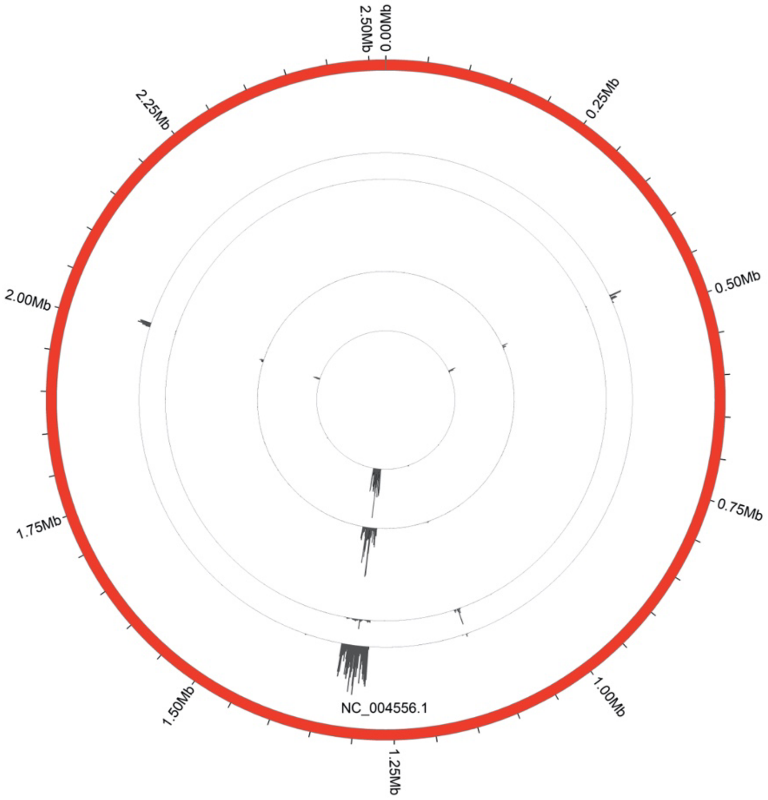

S4: Circo Plot showing coverage of *Xff* Tem1 genome of cellular samples (a) and EV samples (b).

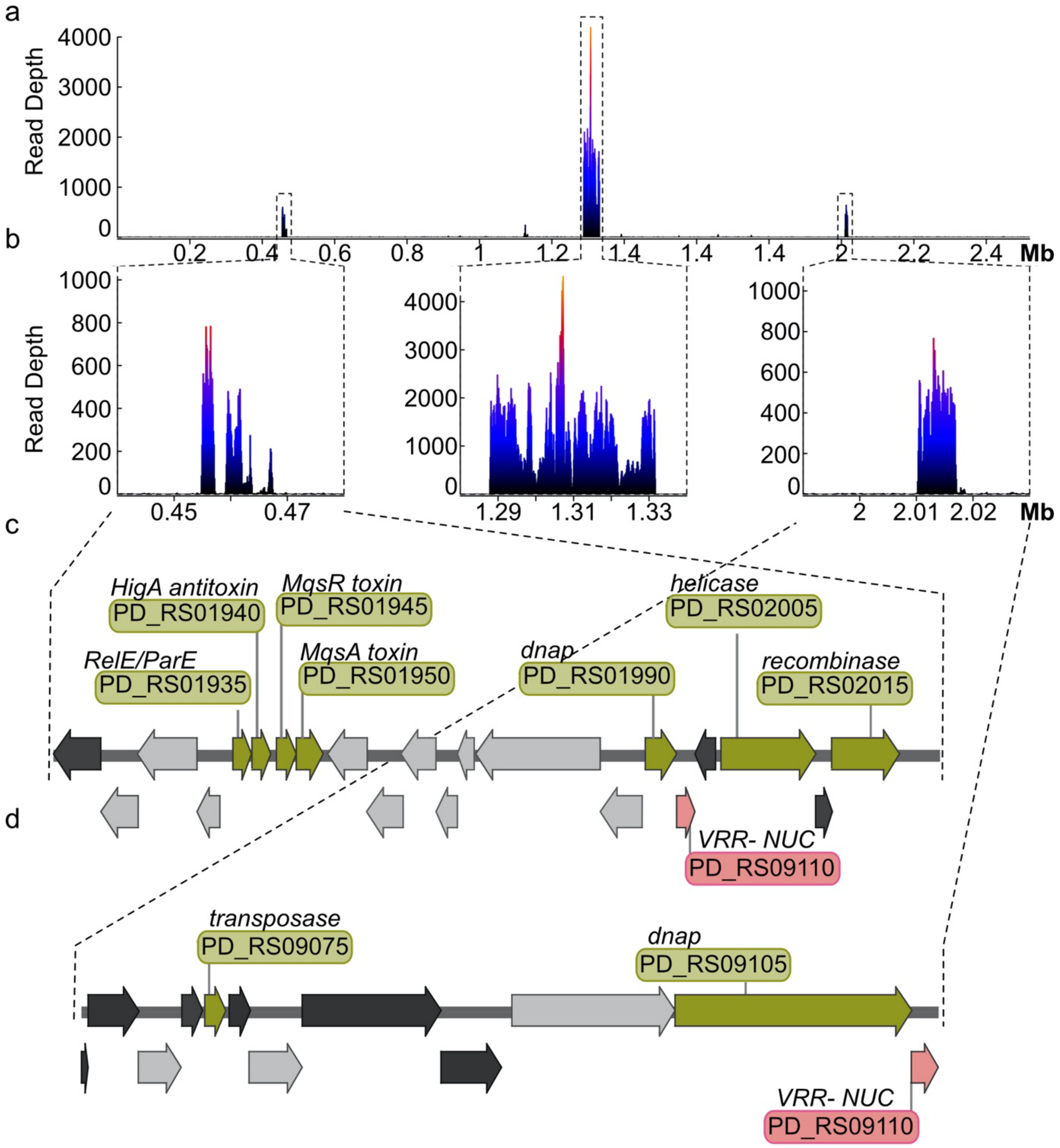

S5: DNA-seq details for region 1 and region 3. Visualization for c) and d) was done using SnapGene software (www.snapgene.com).

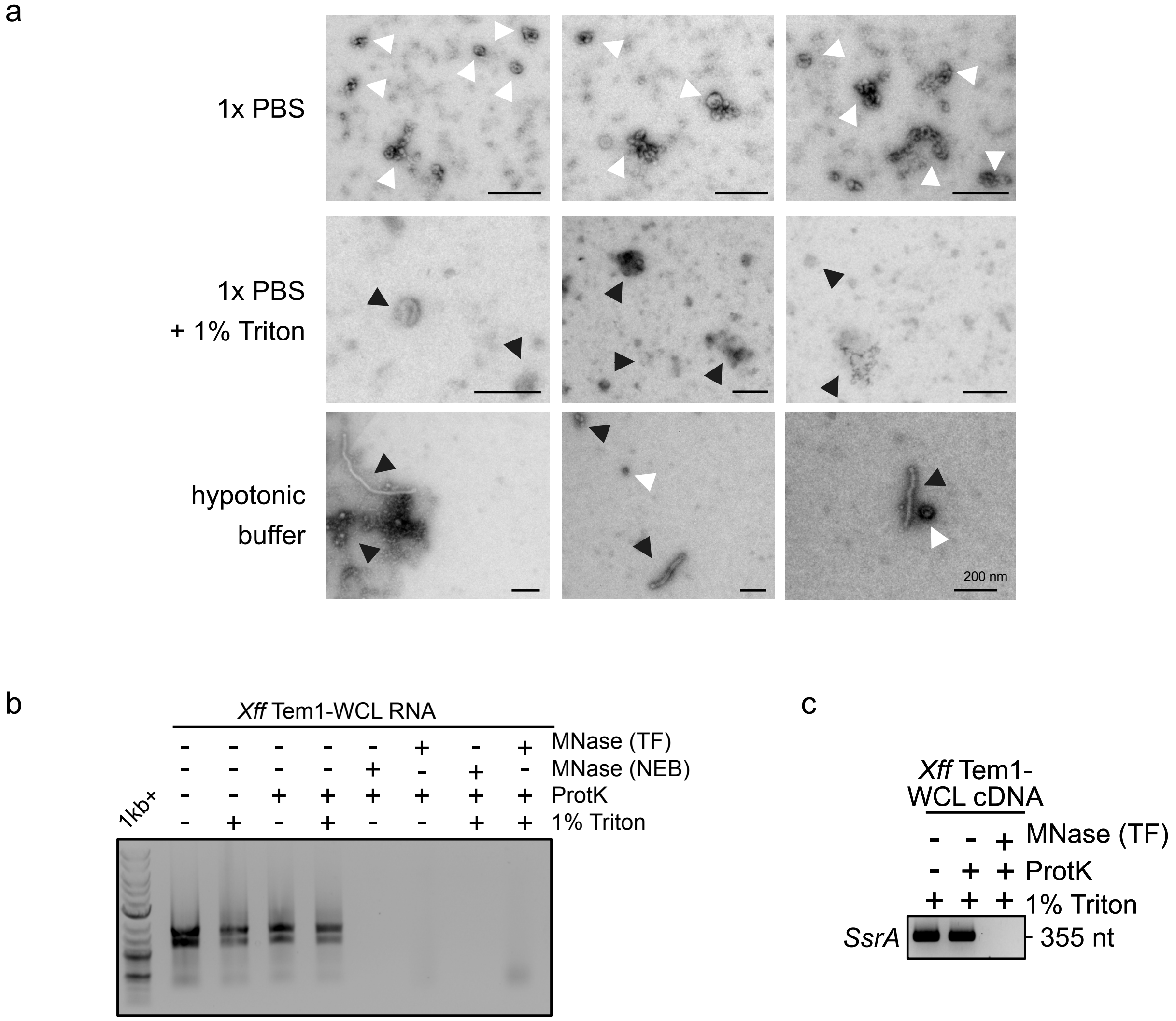

S6: Disruption assays of *Xff* Tem1-EVs. a) Transmission Electron Microscopy with negative staining of *Xff* Tem1-EVs resuspended in 1x PBS or 1x PBS + 1% Triton-X-100 or in hypotonic buffer. White arrow heads point at EV-like structures and black arrow heads at membrane fragments. **b)** ProtK and MNase from two vendors (NEB and ThermoFisher, TF) were tested on naked, cellular RNA from *Xff* Tem1 in the indicated conditions. RNA was visualized on 1% Agarose gel stained with EtBr. **c)** Gene-specific RT-PCR of *SsrA* on cellular RNA, which was enzyme-treated (ProtK alone or co-treatment with MNase) in presence of 1% Triton-X-100, amplified with 40x cycles.

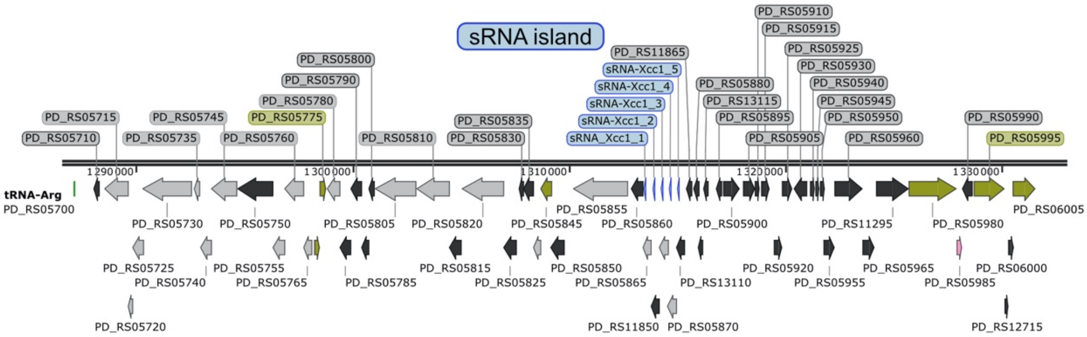

S7: Annotation of genomic island (region 2) with sRNA island containing 5 homologues *sXFs* and the tRNA-Arg gene upstream. Visualization was done using SnapGene software (www.snapgene.com).

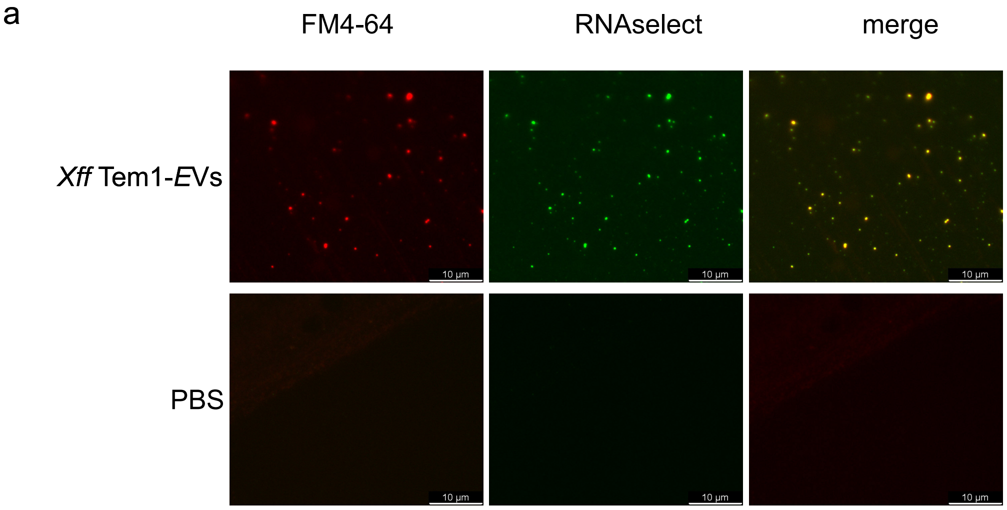

S8: RNAs associate with *Xff* Tem1-EVs. Colocalization of membrane signal (FM4-64) and RNA signal (RNAselect) confirms association of RNA with EVs.
